# Integration of molecular and physiological models to explain time of anthesis in wheat

**DOI:** 10.1093/aob/mct224

**Published:** 2013-11-11

**Authors:** Hamish E. Brown, Peter D. Jamieson, Ian R. Brooking, Derrick J. Moot, Neil I. Huth

**Affiliations:** 1 The New Zealand Institute for Plant & Food Research, Private Bag 4604, Christchurch, New Zealand; 2 21 Melville St, Christchurch 8053, New Zealand; 3 The New Zealand Institute for Plant & Food Research, Private Bag 4442, Palmerston North, New Zealand; 4 Faculty of Agriculture and Life Sciences, PO Box 85084, Lincoln University 7647, Canterbury, New Zealand; 5 CSIRO Ecosystem Sciences, PO Box 102, Toowoomba, Australia 4350

**Keywords:** Anthesis, model, phenology, photoperiod, temperature, short-day vernalization, *Triticum aestivum*, vernalization, *Vrn1*, *Vrn2*, *Vrn3*, *Vrn4*, wheat

## Abstract

**Background and Aims:**

A model to predict anthesis time of a wheat plant from environmental and genetic information requires integration of current concepts in physiological and molecular biology. This paper describes the structure of an integrated model and quantifies its response mechanisms.

**Methods:**

Literature was reviewed to formulate the components of the model. Detailed re-analysis of physiological observations are utilized from a previous publication by the second two authors. In this approach measurements of leaf number and leaf and primordia appearance of near isogenic lines of spring and winter wheat grown for different durations in different temperature and photoperiod conditions are used to quantify mechanisms and parameters to predict time of anthesis.

**Key Results:**

The model predicts the time of anthesis from the length of sequential phases: 1, embryo development; 2, dormant; 3, imbibed/emerging; 4, vegetative; 5, early reproductive; 6, pseudo-stem extension; and 7, ear development. Phase 4 ends with vernalization saturation (VS), Phase 5 with terminal spikelet (TS) and Phase 6 with flag leaf ligule appearance (FL). The durations of Phases 4 and 5 are linked to the expression of *Vrn* genes and are calculated in relation to change in Haun stage (HS) to account for the effects of temperature per se. *Vrn1* must be expressed to sufficient levels for VS to occur. *Vrn1* expression occurs at a base rate of 0·08/HS in winter ‘Batten’ and 0·17/HS in spring ‘Batten’ during Phases 1, 3 and 4. Low temperatures promote expression of *Vrn1* and accelerate progress toward VS. Our hypothesis is that a repressor, *Vrn4*, must first be downregulated for this to occur. Rates of *Vrn4* downregulation and *Vrn1* upregulation have the same exponential response to temperature, but *Vrn4* is quickly upregulated again at high temperatures, meaning short exposure to low temperature has no impact on the time of VS. VS occurs when *Vrn1* reaches a relative expression of 0·76 and *Vrn3* expression begins. However, *Vrn2* represses *Vrn3* expression so *Vrn1* must be further upregulated to repress *Vrn2* and enable *Vrn3* expression. As a result, the target for *Vrn1* to trigger VS was 0·76 in 8-h photoperiods (Pp) and increased at 0·026/HS under 16-h Pp as levels of *Vrn2* increased. This provides a mechanism to model short-day vernalization. *Vrn3* is expressed in Phase 5 (following VS), and apparent rates of *Vrn3* expression increased from 0·15/HS at 8-h Pp to 0·33/HS at 16-h Pp. The final number of leaves is calculated as a function of the HS at which TS occurred (TS^HS^): 2·86 + 1·1 × TS^HS^. The duration of Phase 6 is then dependent on the number of leaves left to emerge and how quickly they emerge.

**Conclusions:**

The analysis integrates molecular biology and crop physiology concepts into a model framework that links different developmental genes to quantitative predictions of wheat anthesis time in different field situations.

## INTRODUCTION

The accuracy in prediction of the growth and development of annual crops depends specifically on the ability to predict the time of the change from vegetative to reproductive growth. This determines the weather conditions in which the crop grows its grain so has a major impact on yield. For wheat, the point that marks the transition from growing vegetative to growing reproductive structures occurs at anthesis. The ultimate anthesis model would simulate the underlying processes that lead to anthesis and provide quantitative estimates of occurrence in any specified environment for any specified genotype by linking genetic information to environmental response coefficients. This would enable rapid characterization of the anthesis behaviour of specific genotypes. It would also enable rapid screening of the adaptive fitness of progeny in a breeding programme by linking molecular markers for development genes/alleles to model coefficients and running simulations to determine the range of anthesis times that will occur in the location for which it is being selected. The phenology and reproductive molecular biology of the wheat crop are well studied because they are complex and provide a guide to the processes of development in other species. However, a model that is suitable for these purposes is yet to be created.

Current wheat anthesis models can be separated into those based on crop physiology or crop molecular biology and biochemistry. Physiological models are incorporated into several accurate wheat simulation models that can predict anthesis when calibrated with appropriate observed data. Genotype differences are handled by using specific ‘genetic’ coefficients to calculate the effects of responses to photoperiod (Pp), temperature, vernalization and inherent earliness. The connection of these coefficients to actual genes is tenuous (White *et al.*, 2008). Molecular anthesis models identify the developmental genes, and describe pathways explaining the interaction of the proteins that these genes express and how these either accelerate or delay progress towards the transition from vegetative to reproductive development (Distelfeld *et al.*, 2009; Trevaskis, 2010; Li *et al.*, 2011). The overall strength of molecular models is their explicit description of the fundamental processes involved in vernalization and Pp responses and their direct linkage of these processes to genetic variation. Their weakness is their lack of a systematic handling of the relationship between gene expression and sequential phenological events, and therefore an inability to give quantitative estimates of anthesis time. In contrast, the weakness of physiological models is their conceptual handling of vernalization and Pp responses. Their strength is their systematic handling of phenological development and therefore their quantitative and consequently predictive ability.

In this paper we develop a quantitative model of the expression of specific developmental genes and combine it with aspects of physiological models that predict anthesis time in response to the environment. First, we review literature to hypothesize the structure of the model. We then re-analyse the large dataset of physiological measurements from Brooking and Jamieson (2002) to infer the behaviour of genes and the products they express.

### Environmental responses and genetic variation

The inverse of the time to anthesis is the average development rate. Wheat development demonstrates two contrasting responses to temperature (Porter and Gawith, 1999). High temperatures usually accelerate development (temperature per se), but in some genotypes a period of low temperature may also hasten development (vernalization). Wheat also demonstrates two contrasting Pp responses; short days may accelerate development during vernalization (Evans, 1987; Brooking and Jamieson, 2002; Allard *et al.*, 2012), but delay development following vernalization (Brooking *et al.*, 1995; Slafer and Rawson, 1996). These responses have evolved to enable wheat seeds to germinate across a wide range of calendar dates but to flower over a narrower range to ensure grain is grown when environmental conditions are most favourable for reproductive success. This characteristic was described by Hay and Kirby (1991) as ‘convergence’. Wheat shows considerable genetic variation in environmental responsiveness with some types that respond only to temperature per se, some that also respond to Pp, some that have an additional vernalization response to low temperature and some that also show Pp effects in vernalization response (Hay and Kirby, 1991). Within types of wheat there is also variation in the extent of environmental response (Syme, 1973; Halloran, 1975; Slafer and Rawson, 1995*b*; van Beem *et al.*, 2005; Trevaskis *et al.*, 2007; Distelfeld *et al.*, 2009; Eagles *et al.*, 2010; Yang *et al.*, 2011): (1) to temperature per se, called earliness per se; (2) to low temperatures, called vernalization sensitivity; and (3) to Pp following vernalization, called Pp sensitivity. The range of types and sensitivities has arisen as wheat has adapted to achieve ‘convergence’ to the optimal anthesis time for a broad range of environments.

### A brief description of wheat phenology

We describe current knowledge of wheat development within the ‘Kirby Framework’, named in honour of the late E. J. M. Kirby, who originated the concepts. A particularly important concept is that of ‘synchrony’, where the development of later tillers is synchronized with the development of the main stem, so that any developmental study can concentrate on the development of the main stem alone. Development on the main stem proceeds by the accumulation of primordia on the apical meristem, their development into physical structures, the extension of the true stem up through the enveloping leaf sheaths, the emergence of the ear, anthesis (through grain filling) and physiological maturity (Kirby, 1990, 1993; Kirby *et al.*, 1999). The appearance of leaves on the main stem is a linear function of temperature (Kirby, 1995) from close to 0 °C to at least 30 °C (Jamieson *et al.*, 2008), but may peak and then reduce with increasing temperatures above 30 °C (Parent and Tardieu, 2012). The inverse of the leaf appearance rate is the phyllochron, the interval between the appearance of successive leaves, and this differs among genotypes (Jamieson and Munro, 2000). The coordination of primordium production with leaf appearance is such that the latter provides a useful timeframe for analysing the former (Kirby, 1990; Brooking and Jamieson, 2002), especially when current leaf number is expressed using the decimal Haun stage (HS; Haun, 1973) to give more precision – effectively defining ‘developmental time’. When this is done graphically, a major feature that is obvious is that the primordium production rate accelerates following vernalization saturation (VS) when vernalization requirements are satisfied (Hay and Kemp, 1990; Kirby, 1990; Brooking, 1996; Brooking and Jamieson, 2002). Thus, primordium development can be divided into two stages, where primordia are produced at either the lower ‘vegetative’ rate or the higher ‘reproductive’ rate. Vernalization and Pp responses are expressed through changes in the final number of leaves (FLN) produced on the main stem (Brooking *et al.*, 1995; Brooking and Jamieson, 2002). As FLN increases, the difference between the ‘vegetative’ and ‘reproductive’ rates of primordium production reduces so that, when FLN becomes large, the two rates are nearly indistinguishable (Brooking and Jamieson, 2002). In this paper, we refer to the time at which the rate change takes place as floral initiation (FI), although this does not mean that all the following primordia will become floral organs. The non-visible event of VS occurs either at the same time or at some time prior to the observable event of FI.

The number of primordia produced at the time of FI does not necessarily correlate with the final number of leaves because at this time there are a number of primordia that may become either leaves or spikelets (Griffiths *et al.*, 1985; Brooking and Jamieson, 2002). There are two more subsequent events on the apex that are important to determine the ultimate fate of primordia:

1. the formation of double ridges (DR), described by Baker *et al.* (1986) as the first unequivocal sign that a primordium has committed to a reproductive fate;
2. the formation of the last or terminal spikelet (TS) primordium, which signals the halting of further primordium production.

DR formation is first observable on primordia in the middle of the spike and a wave of commitment to reproductive fate moves up and down the spike from this point. Between DR and TS there are a number of primordia, between those already displaying double ridges in the centre of the spike and those committed to becoming leaves on the lower part of the apex, whose fate is not yet determined. These so-called labile primordia could become either leaves or spikelets (Griffiths *et al.*, 1985; Brooking and Jamieson, 2002). Jamieson *et al.* (2007) showed that the HS at which TS occurred (TS^HS^) is tightly related to FLN, using data derived from many environments (both field and controlled environment) and genotypes. Sonego *et al.* (2000) found similar relationships for TS in oats. This suggests that the commitment of primordia to becoming leaves moves up the stem at a rate that is constant relative to leaf appearance and meets the wave of reproductive commitment coming down the apex at the time of TS. Thus, environmental cues that determine the rate of progress of the wave of commitment and the timing of TS will determine FLN. Once the wheat plant reaches TS, all the leaves that are committed to a vegetative fate must appear before the appearance of the flag leaf ligule (FL) and subsequent anthesis occurs. Overall, the time of anthesis is controlled by the effects of vernalization and Pp on FLN, and the effects of temperature and phyllochron on the time it takes for the FL to appear.

### Physiological models of time to flower for wheat

Physiological models of time to anthesis incorporate calculations of the effect temperature and Pp have on progress to anthesis. Experimental studies that underlie physiological models are conducted in the field or controlled environment facilities. The observations required are relatively easy to collect so experiments usually apply a number of levels of treatments to quantify response profiles and/or take repeated measures of a range of variables (e.g. primordium initiation, leaf appearance, flag leaf appearance) that explain the progress toward anthesis. Physiological models are widely used to predict the time to flower and are useful tools in the study of the genetic controls of crop processes (Hammer *et al.*, 2006; Yin and Struik, 2010; Allard *et al.*, 2012). In spite of the potential that physiological models offer in advancing genetic studies, success in linking genetic coefficients to genotype information has been limited (White *et al.*, 2008).

We review the two main successful approaches incorporated into the most widely used wheat simulation models. The differences in these approaches represent the evolution of understanding of the process, rather than competing theories of development. What the approaches have in common is the use of a developmental timescale, the way the plant sees time. This is based on the concept of thermal time (Tt), calculated by summing the accumulation of daily temperature above a base temperature below which development ceases.

The CERES group of models (Ritchie and Otter, 1985) and ARCWHEAT1 (Weir *et al.*, 1984) calculate phenological progress through a succession of phases between FI, DR (ARCWHEAT1) and TS (CERES and ARCWHEAT1) driven by the accumulation of modified Tt. Anthesis occurs a fixed amount of Tt beyond TS. Vernalization (Vf) and Pp (Pf) factors (with values between zero and unity) reduce the rate of accumulation of Tt so that exposure to short days and incomplete vernalization lead to an increase in the amount of unmodified Tt required to reach anthesis. Vernalization is treated as a cumulative process, with a base amount of vernal time (Vt) required to lift Vf above zero, and a saturation Vt to bring Vf to unity. There is a function that relates vernalization effectiveness with temperature, so that Vt accumulates at 1 unit per day between 4 and 10 °C, but reduces linearly to zero at temperatures above 16 °C and below –4 °C in ARCWHEAT1. A similar function is used in the CERES models, but the details differ (McMaster *et al.*, 2008). Pf increases with Pp up to a Pp that is assumed to be long enough to maximize development. Implementation differs between the CERES and ARCWHEAT1 models, and among different versions and derivatives of the CERES Wheat models. The CERES models use TS as their intermediate event, so Vf and Pf cease to be applied after that event (McMaster *et al.*, 2008). In ARCWHEAT1, Vf is applied only until FI, and Pf until TS. Differences in genotypes are simulated through vernalization and photoperiod sensitivity coefficients that determine the nature of the response of Vf and Pf to temperature and Pp, respectively. Intrinsic earliness is associated with the modified thermal duration of phase lengths. In these models the thermal phase durations are assumed to be independent. However, Jamieson *et al.* (2007) showed that phase lengths are not independent.

A more complete understanding of phenological development is incorporated into the wheat model Sirius (Jamieson *et al.*, 1998*b*; He *et al.*, 2012). SIRIUS phenology is based on the ‘Kirby framework’, which provides a link between vegetative development (leaf appearance) and reproductive development (final leaf number). Development progress before emergence and after FL is based on Tt accumulation and Tt targets. The phase between emergence and FL is based on leaf appearance (using the HS) and the FLN target (Jamieson *et al.*, 1998*b*). Responses to Pp and temperature are used to vary main stem FLN. Thus, the timing of FL depends upon FLN, phyllochron and the accumulation of Tt (Jamieson *et al.*, 1998*a*). Phyllochron is calculated in Tt so it captures the effect of temperature per se and varietal differences in its value quantify genetic variation in earliness per se (Jamieson *et al.*, 1998*a*; Jamieson and Munro, 1999). Considerable effort has been put into determining the correct temperature for calculating Tt accumulation and quantifying the pattern of phyllochron throughout the duration of leaf appearance (Jamieson *et al.*, 1995, 2008). The mechanisms SIRIUS uses to calculate FLN were described by He *et al.* (2012). Briefly, for winter wheat types the potential FLN begins at a high value (19–26) and exposure to cold conditions causes this to decline in proportion to an accumulated vernalization index. The temperature that facilitates the fastest reduction in FLN is 8 °C and the rate decreases to have no effect at 0 and 15 °C. Primordium number (produced at the ‘vegetative rate’) is estimated from its relationship with HS. Vernalization is complete when one of three conditions is met: the vernalization index reaches 1, the potential FLN decreases to about 8 or the increasing primordium number reaches the decreasing potential FLN. The primordium number at this stage represents the FLN that would occur under long Pp conditions. Pp responses are modelled by adding to the FLN determined at the completion of vernalization. As spring wheat types have a small vernalization requirement, they always have a small potential FLN (6–9) and Pp responses occur as soon as the crop is competent to perceive Pp stimulus (Brooking *et al.*, 1995). Shorter Pp facilitate an increase in the number of leaves that are added to FLN and this increases linearly from 0 at Pp above 16 h to a maximum at Pp below 8 h. The actual number of leaves to be added is determined on the day that the current HS is exactly half the FLN calculated from the current Pp. This target changes each day so is recalculated daily and FLN is committed on the day that the target is reached. An important effect of this is that the calculated FLN increases each day as Pp declines, effectively delaying anthesis, while calculated FLN decreases each day as Pp increases. Therefore, autumn and spring sowings of the same genotypes will have different FLN even if they emerge in similar Pp, and their anthesis dates will ‘converge’.

### Molecular models of anthesis in wheat

Molecular models of anthesis describe the pathways of gene expression required for anthesis and the effects of temperature and Pp on this gene expression (Distelfeld *et al.*, 2009; Trevaskis, 2010; Li *et al.*, 2011). These models advance fundamental understanding of genetic and environmental control of anthesis. The experimental work for these models is usually conducted in the laboratory or glasshouse. It focuses on the creation and characterization of individuals with small differences in the genetic construction and the effect of these differences on gene expression. Because of the technical complexities in isolating, cloning, sequencing and transforming the genetics of individuals and measuring the expression of genes, experimental work usually has few levels of environmental treatment and few repeat observations. The small amount of plant material to work with means simple measures of phenotype, such as days to heading or the state of the apex following a treatment, are used. As a consequence, the molecular models are qualitative, describe fundamental processes and how genotype influences these, but do not provide a prediction of the time to flower.

Molecular models are not as easily defined as physiological models because they are not usually implemented into executable code and do not assume the same ‘brand’ identity that physiological models have. Rather, molecular models provide a qualitative articulation of the results of complex molecular analysis and an explanation of how a genetic or environmental factors influences an outcome. Molecular models of anthesis timing have been developed over the last 10 years as authors build on previously published results (Dubcovsky *et al.*, 2006*b*; Yan *et al.*, 2006; Hemming *et al.*, 2008; Greenup *et al.*, 2009; Sasani *et al.*, 2009; Shimada *et al.*, 2009; Distelfeld and Dubcovsky, 2010; Trevaskis, 2010; Li *et al.*, 2011). All molecular models contain *Vrn1*, *Vrn2* and *Vrn3* (often called FT) as the key genes that control the time to anthesis and agree upon the nature of their expression in response to environment. Genetic variation and environmental signals control the rate at which these genes express proteins. Genes are referred to using italics (e.g. *Vrn1*), the proteins they express in plain text (e.g. Vrn1), the amount of protein expressed is represented as a concentration of these proteins (e.g. [Vrn1]) and the rate of expression as a change in the concentration (e.g. Δ[Vrn1]). The terms up and down regulation refer to an increase or decrease in [Vrn]. The terms promotion and repression refer to an increase or decrease in the rate that a protein is transcribed by its gene which represents an increase or decrease in Δ[Vrn1]. Vrn1 is present in low concentrations in young plants and [Vrn1] increases over time in both spring and winter types (Trevaskis *et al.*, 2007; Diaz *et al.*, 2012). For winter genotypes of *Vrn1*, Δ[Vrn1] is promoted under cold conditions whereas spring genotypes have a high Δ[Vrn1] regardless of temperature. Vrn1 controls observed vernalization responses in wheat and barley (Trevaskis *et al.*, 2006, 2007; Hemming *et al.*, 2008; Sasani *et al.*, 2009; Shimada *et al.*, 2009). Vrn3 is observed only in the presence of Vrn1, Δ[Vrn3] is promoted by longer Pp, and genetic variations in *Vrn3* and *Ppd1* control Δ[Vrn3] at a given Pp and infer observed Pp sensitivities (Whitechurch and Slafer, 2002; Dubcovsky *et al.*, 2006*b*; Tanio and Kato, 2007; Sasani *et al.*, 2009; Guo *et al.*, 2010; Yang *et al.*, 2011; Shaw *et al.*, 2012; Kitagawa *et al.*, 2012; Allard *et al.*, 2012). [*Vrn2*] is also upregulated with increased Pp (Karsai *et al.*, 2005; Dubcovsky *et al.*, 2006*b*; Trevaskis *et al.*, 2006; Sasani *et al.*, 2009) and *Vrn2* interacts with *Vrn1* and *Vrn3* (Li *et al.*, 2011). A Vrn4 locus has also been mapped (Yoshida *et al.*, 2010) but is yet to be cloned. The role of Vrn4 in controlling anthesis is not fully understood and is omitted from the model of Li *et al.* (2011). However, Yoshida *et al.* (2010) have presented results suggesting *Vrn4* affects responses to short vernalization periods.

The most comprehensive model explaining the interactions of *Vrn1*, *Vrn2* and *Vrn3* was presented by Li *et al.* (2011). In their model, Vrn1 is the protein that triggers transition of the apex from vegetative to reproductive behaviour (Trevaskis, 2010) that culminates in the occurrence of TS. The activity of *Vrn1* alone will not express sufficient [Vrn1] to trigger TS. Vrn3 is a promoter that increases Δ[Vrn1] to give sufficient [Vrn1] to trigger TS. Vrn3 is only observed in the presence of Vrn1, so a certain [Vrn1] is required to promote Δ[Vrn3] which promotes transcription of *Vrn1* giving subsequent increases in [Vrn1]. The occurrence of sufficient [Vrn1] to promote Δ[Vrn3] could represent VS as Δ[Vrn3] is Pp sensitive, and physiological observations show the crop becomes Pp sensitive following VS. Vrn2 represses the expression of *Vrn3* which stops the occurrence of VS. Vrn1 in turn represses *Vrn2*. So the presence of Vrn2 delays VS because higher [Vrn1] will be required before Vrn3 can be expressed and Pp responses become apparent. This model is also consistent with physiological observations where vernalization responses (a reduction in FLN in response to low temperature or short photoperiod) occur first followed by photoperiod responses (an increase in FLN in response to short photoperiod). Specifically, the interaction between Vrn1 and Vrn2 as a precursor to the expression of Vrn3 constitutes a vernalization response followed by Vrn3, further upregulating Vrn1, which constitutes the subsequent Pp response.

### Potential for model improvement

Hay and Ellis (1998) predicted that the rapid advances being made regarding the molecular controls of anthesis would help to improve cereal models. However, to date, there have been few changes to existing crop physiological models based on insights gained at the molecular level. The limited success shown in linking the genetic coefficients of physiological models to wheat anthesis time genotypes (White *et al.*, 2008) suggests current physiological models do not accurately represent all of the underlying molecular processes that control anthesis. For instance, the CERES vernalization and Pp responses are predicted as concurrent events whereas they are actually sequential (Purvis, 1934; Hay and Ellis, 1998; Brooking and Jamieson, 2002; Allard *et al.*, 2012). SIRIUS treats the two processes as sequential events (Jamieson *et al.*, 1998*b*) but still has a number of shortcomings. Specifically, it has no mechanism to account for short-day vernalization (Evans, 1987; Brooking and Jamieson, 2002; Allard *et al.*, 2012) or the effects of temperature on the extent of Pp response (Slafer and Rawson, 1995*c*, 1996; Yan and Wallace, 1996). Further, when we consider controlled environment experiments where plants are grown in short Pp conditions for different durations, then moved to long Pp conditions, SIRIUS fixes FLN on a specific day so will predict a step change in FLN for treatments that were still in short-day conditions when FLN was fixed. However, observed results from such experiments show a gradual increase in FLN with increased exposure to short-day conditions (Slafer and Rawson, 1995*a*; Brooking and Jamieson, 2002; Miralles *et al.*, 2003). Furthermore, both CERES and SIRIUS assume vernalization response begins and increases following any exposure to cold conditions. However, a number of datasets show a lag period of up to 45 d exposure to cold conditions before a reduction in FLN occurs (Wang *et al.*, 1995*a*, *b*; Brooking, 1996; Brooking and Jamieson, 2002). These shortcomings may be overcome by making further refinements to existing SIRIUS mechanisms. However, the model would still not relate well to the fundamental molecular processes that underlie reproductive development and so fail to provide a clear link between anthesis genotype and development behaviour.

### An integrated model

Our aim is to construct a developmental model that integrates assumptions from physiological and molecular models, and in doing so provide a quantitative link between anthesis genotype and anthesis time in a specific environment. Such a model must use current knowledge of the quantitative nature of gene expression to produce behaviour, similar to that of the most accurate current simulation models (and of experimental observations), while identifying the prime causes. Although there are shortcomings in the SIRIUS model, it contains many sound concepts that can form assumptions for the creation of an integrated model:

1. Anthesis occurs at a predictable Tt target beyond flag leaf appearance.
2. Flag leaf appearance date is a function of FLN and leaf appearance rate.
3. Leaf appearance rate can be predicted from phyllochron and Tt.
4. FLN can be predicted from the HS at which TS occurs (Jamieson *et al.*, 2007).

Variation in the timing of TS is caused by vernalization and Pp responses. To create a link between physiological and molecular models, we assume that the progression through phases is triggered by the adequate expression of controlling genes. Vrn1 is recognized as the central control signal in triggering anthesis (Trevaskis, 2010) so we assume:

5. FI and subsequent TS will occur when [Vrn1] in the stem apex is sufficient.
For the mechanisms controlling Δ[Vrn1], we draw on the model presented by Li *et al.* (2011) and assume
6. A certain [Vrn3] is required to upregulate [Vrn1] to adequate levels to trigger FI and TS.
7. Vrn2 represses the expression of *Vrn3*.
8. Vrn1 represses the expression of *Vrn2*.
9. VS occurs when [Vrn1] is sufficient to repress Δ[Vrn2] and promote Δ[Vrn3].

These assumptions provide a mechanism to control anthesis time, but we still require mechanisms for the effects of environmental and genetic factors on progress through this pathway. Photoperiod and vernalization responses can be assumed to be the result of environmental signals repressing or promoting the expression of development genes:

10. Δ[Vrn2] and Δ[Vrn3] are promoted by longer Pp and the extent of this response is dependent on variation (allelic and/or copy number) in *Vrn2*, *Vrn3* and photoperiod-sensing (e.g. *Ppd* and *CO*) genes.
11. Δ[Vrn1] is repressed under high temperatures and the extent of this repression is dependent on variation (allelic and/or copy number) in *Vrn1* genes.

These assumptions provide mechanisms for describing genotypic differences in Pp and vernalization responses. However, there is an additional developmental response to temperature, that of temperature per se on development. Based on the notion that temperature will affect the kinetics of expression rates of all genes (Yan and Wallace, 1996), we assume:

12. Expression of genes described in Assumptions 10 and 11 will be greater at higher temperatures.

Note that the temperature response described in Assumption 11 is in the opposite direction to that of Assumption 12, but the two responses will combine to give a complex overall temperature response. The assumptions presented are based on the literature reviewed. Further elucidation and quantification of the response mechanisms are required to bring these assumptions into a working model. The aim of this paper is to provide these quantifications.

## METHODS

### Experimental data

One of the most detailed studies into the effects of environment on developmental progress of wheat was reported by Brooking and Jamieson (2002). The authors drew a number of important interpretations from their analysis regarding the internal relationship between HS and primordium number and the nature of vernalization responses. However, the information regarding the timing of FI and TS, the photoperiod response of the spring isoline, and a holistic synthesis of all the treatments and observations remained for further analysis. The methods were reported in detail by Brooking and Jamieson (2002). In brief, the experiment used near isogenic lines of ‘Batten’ wheat that differed only in their vernalization requirement and were termed spring and winter isolines (differing in *Vrn1A*). Each isoline was grown for an initial treatment period in a range of cool and/or short Pp conditions that were expected to change FLN. They were then moved into control conditions that were not expected to give any vernalization-related reduction or Pp-related increase in FLN. Initial conditions were the main treatments and included 5, 8 and 11 °C at 16-h Pp, 5, 8, 11 and 23 °C at 8-h Pp and 1 °C in the dark. Final control conditions were 23 °C and 16-h Pp because these conditions were expected to minimize the FLN in spring wheat and maximize it in winter wheat. Within each main treatment, 8–10 sub-treatments were imposed which consisted of different transfer times from an initial treatment to final control conditions. In most cases, transfer time treatments were 7 d apart and ranged from 0 d after sowing (i.e. controls when all the growth cycle was spent at 23 °C and 16-h Pp) to 84 d. Within each of these treatments, destructive measurements were made at 7-d intervals, sampling four plants per treatment to observe the number of primordia and HS. The FLN was also measured once the flag leaf had appeared on all remaining plants (10–20 plants per treatment).

### The relationship between temperature and leaf appearance

**Fig. 1.**
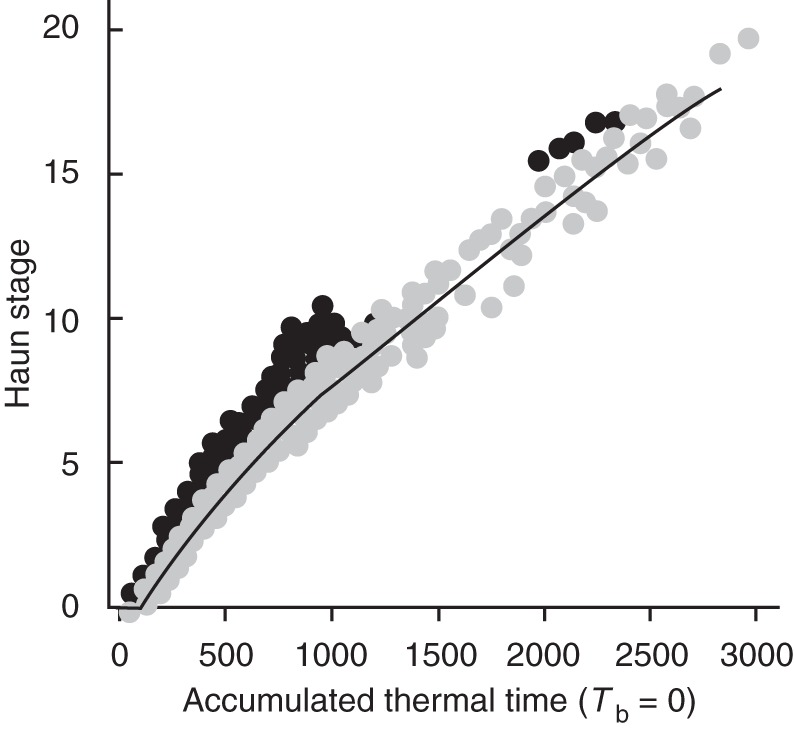
Haun stage against accumulated Tt above base temperature (*T*_b_) of zero for ‘Batten’ wheat. Black symbols represent the treatments that were vernalized at 11 °C and the grey symbols represent all other treatments.

Our analysis required the determination of HS at the time of FI and TS, and this often occurred on dates between the observations of HS. To enable complete analysis we estimated HS at the necessary times from the relationship between HS and accumulated Tt. Jamieson *et al.* (1998*b*) calculated HS in relation to Tt accumulated above a base temperature of 0 °C assuming it takes one phyllochron for emergence to occur. Following emergence, the phyllochron is 75 % of the genotypes base phyllochron until HS 2·0, 100 % of base phyllochron until HS 8·0 and then 140 % of base phyllochron for HS later than 8·0 (Jamieson *et al.*, 1995, 1998*b*). The applicability of this relationship for the experimental dataset (Fig. 1) suggested minor modifications were required. The base phyllochron was increased to 120 °Cd, emergence took 0·9 phyllochron, and the break points for change in phyllochron were positioned at 2·5 and 7·0 HS.

The treatments that did not follow this relationship (Fig. 1) were those that had initial treatment temperatures of 11 °C. Closer investigation of this response (not shown) indicated there was no discrepancy for the 0 d transfer sub-treatment, but the discrepancy increased as treatments were exposed to longer durations of the 11 °C conditions. This suggests that the temperature these plants encountered was actually higher than 11 °C, or, less likely, the phyllochron was different in the 11 °C treatments. An increase of the presumed temperature of this treatment to 16 °C was required for it to conform to the other treatments. Brooking and Jamieson (2002) ensured apex temperatures were close to those set in the growth rooms. However, measurements of actual temperatures were only taken at the very beginning of the 11 °C treatment cycle so the possibility the temperatures were higher than 11 °C cannot be ruled out. To conduct the intended analysis we needed estimates of the HS on specific days. For these calculations it was assumed the temperature in the 11 °C treatments was 16 °C to enable correct estimations of HS. To quantify the temperature response we need to know the temperature of this treatment. However, as this discrepancy cannot be resolved, we will plot results for this treatment against the range of possible temperatures so judgments can be made about the effect of this uncertainty on the interpretations drawn.

### Estimating Haun stage of FI and TS

**Fig. 2.**
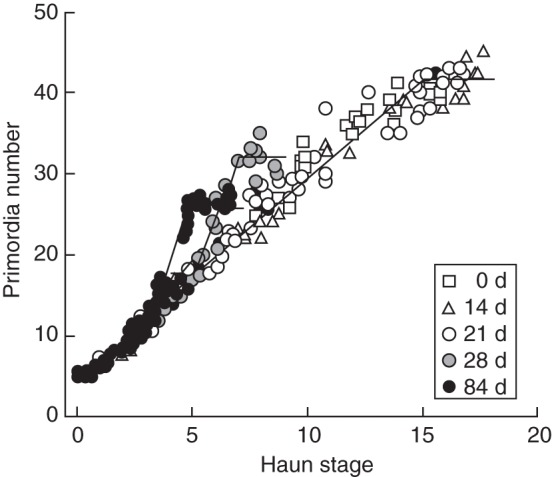
An example of the relationship between primordium number and Haun stage fitted to 0-, 14-, 21-, 28- and 84-d transfer times for ‘Batten’ winter wheat in the 8 °C, 16-h Pp treatment. Lines fitted to the 84- and 28-d sub-treatments separately and a base relationship is fitted to all treatments that had a FLN of 19.

Trilinear relationships were fitted by eye to all individual plots of cumulative primordia versus HS as shown in Fig. 2. The HS at which FI occurred (FI^HS^) was defined as the HS at which the number of primordia differed from the base relationship shown in Fig. 2. The TS^HS^ was determined as the HS when no further increase in the number of primordia occurred (Fig. 2). Fitted TS^HS^ and FI^HS^ values are displayed in Table 1 for the spring isoline and Table 2 for the winter isoline. For the winter isoline, there were a number of treatments where insufficient data prevented a reliable estimate of TS^HS^ and the primordium initiation rate did not depart from the base relationship so no estimate of FI^HS^ could be obtained. It was important for the analysis to have a value for FI^HS^ and TS^HS^ for each treatment, so these stages were estimated for treatments where it could not be fitted. First, the TS^HS^ was estimated from its relationship with FLN because FLN was reliably measured. Jamieson *et al.* (2007) showed a strong relationship between TS^HS^ and FLN. We plotted all data (not shown) and also obtained a strong (*R*^2^ > 0·95) linear relationship, FLN = 2·86 + 1·1TS^HS^. This was re-arranged to estimate TS^HS^ from FLN.

**Table 1.** The Haun stage (HS) at transfer (T), floral initiation (FI) and terminal spikelet (TS) for the spring isoline of ‘Batten’ wheat treated with different temperature (°C) and Pp (h) conditions for differing periods of time before transfer into 23 °C, 16-h conditions for the remainder of the experiment

Treatment	HS	Transfer (d after sowing)													
		0	7	14	21	28	35	42	49	52	56	63	67	70	84
1 °C, 0 h	T	–	–1·1	–1·1	–1·0	–0·9	–0·8	–0·8	–0·7	–	–0·6	–	–0·5	–	–0·3
	FI	–	3·6	3·4	3·2	3·0	3·0	3·0	3·0	–	3·0	–	3·0	–	3·0
	TS	–	6·0	6·3	6·0	5·8	5·8	5·8	5·8	–	5·8	–	5·8	–	5·8
5 °C, 8 h	T	–1·2	–0·8	–0·4	–0·1	0·3	0·7	1·1	1·5	–	–	2·3	–	–	3·2
	FI	2·5	2·5	2·5	2·5	2·5	2·7	3·1	3·2	–	–	3·5	–	–	3·6
	TS	4·8	4·8	4·8	4·8	4·8	5·1	5·0	5·3	–	–	5·5	–	–	5·6
5 °C, 16 h	T	–	–0·8	–0·4	–0·1	0·3	0·7	1·1	–	–	1·9	–	–	2·6	**3·2**
	FI	–	3·5	3·5	3·5	3·3	3·3	3·0	–	–	2·7	–	–	2·5	**2·5**
	TS	–	5·7	5·7	5·7	5·3	5·3	5·0	–	–	4·7	–	–	4·7	**4·7**
8 °C, 8 h	T	–	–	0·0	0·6	1·3	1·9	2·5	3·0	–	3·4	–	–	**4·3**	–
	FI	–	–	2·3	2·7	3·0	3·3	3·3	3·3	–	3·3	–	–	**3·3**	–
	TS	–	–	4·7	4·9	5·0	5·3	5·6	5·7	–	6·0	–	–	**6·5**	–
8 °C, 16 h	T	–1·2	–	0·0	0·6	1·3	1·9	2·5	–	**3·2**	–	**3·9**	–	–	**5·3**
	FI	2·7	–	2·7	2·6	2·3	2·3	2·3	–	**2·3**	–	**2·3**	–	–	**2·3**
	TS	4·7	–	4·7	4·7	4·5	4·3	4·3	–	**4·3**	–	**4·3**	–	–	**4·3**
11 °C, 8 h	T	–1·2	0·0	1·3	2·5	3·4	4·3	**5·3**	**6·2**	–	**7·1**	–	–	–	**9·7**
	FI	3·5	3·5	3·9	4·4	4·5	4·5	**4·5**	**4·5**	–	**4·5**	–	–	–	**4·5**
	TS	5·5	5·5	5·8	6·1	6·8	7·0	**7·3**	**7·7**	–	**8·0**	–	–	–	**8·8**
11 °C, 16 h	T	–1·2	0·0	1·3	2·5	**3·4**	**4·3**	**5·3**	**6·2**	–	**7·1**	**7·7**	–	–	–
	FI	3·5	3·3	2·9	2·5	**2·5**	**2·5**	**2·5**	**2·5**	–	**2·5**	**2·5**	–	–	–
	TS	6·3	5·7	4·9	4·5	**4·5**	**4·5**	**4·5**	**4·5**	–	**4·5**	**4·5**	–	–	–
23 °C, 8 h	T	–	0·6	2·4	**3·8**	**5·1**	**6·4**	**7·6**	**8·5**	–	–	–	–	–	**13·4**
	FI	–	3·6	4·0	**4·5**	**4·5**	**4·5**	**4·5**	**4·5**	–	–	–	–	–	**4·5**
	TS	–	5·6	6·2	**6·6**	**7·5**	**8·0**	**8·5**	**9·0**	–	–	–	–	–	**9·0**

Values in bold text are for treatments where transfer occurred after FI and bold underlined text are treatments where transfer occurred after TS.

**Table 2.** The Haun stage (HS) at transfer (T), floral initiation (FI) and terminal spikelet (TS) for the winter isoline of ‘Batten’ wheat treated with different temperature (°C) and Pp (h) conditions for differing periods of time before transfer into 23 °C, 16-h conditions for the remainder of the experiment

Treatment	HS	Transfer (d after sowing)													
		0	7	14	21	28	35	42	49	52	56	63	67	70	84
1 °C, 0 h	T	–	0·0	0·0	0·0	0·0	0·0	0·0	0·0	–	0·0	–	0·0	–	0·0
	FI	–	*12·2*	*12·4*	*12·1*	*12·0*	*12·7*	*12·5*	*11·6*	–	10·0	–	7·5	–	4·5
	TS	–	*14·2*	*14·4*	*14·1*	*14·0*	*14·7*	*14·5*	*13·6*	–	12·0	–	9·5	–	6·5
5 °C, 8 h	T	0·0	0·0	0·0	0·0	0·2	0·6	1·0	1·4	–	–	2·2	–	–	3·1
	FI	*13·3*	*13·3*	*13·5*	*12·4*	8·0	3·8	3·8	3·5	–	–	3·7	–	–	3·7
	TS	*15·3*	*15·3*	*15·5*	*14·4*	10·0	6·2	5·4	5·2	–	–	5·4	–	–	5·4
5 °C, 16 h	T	–	0·0	0·0	0·0	0·2	0·6	1·0	–	–	1·8	–	–	2·5	**3·1**
	FI	–	*13·5*	*13·5*	*12·7*	*12·3*	8·3	4·2	–	–	3·2	–	–	2·5	**2·5**
	TS	–	*15·5*	*15·5*	*14·7*	*14·3*	10·5	7·0	–	–	5·4	–	–	5·2	**5·2**
8 °C, 8 h	T	–	–	0·0	0·5	1·2	1·8	2·4	2·9	–	3·4	–	–	**4·3**	–
	FI	–	–	*13·0*	7·3	4·3	4·2	4·0	4·2	–	3·7	–	–	**3·5**	–
	TS	–	–	15·0	9·5	6·3	6·0	5·8	6·3	–	6·1	–	–	**6·3**	–
8 °C, 16 h	T	0·0	–	0·0	0·5	1·2	1·8	2·4	–	3·1	–	**3·8**	–	–	**5·2**
	FI	*11·4*	–	*11·9*	*11·0*	5·0	4·2	3·2	–	3·2	–	**3·0**	–	–	**3·0**
	TS	*13·4*	–	*13·9*	*13·0*	7·0	6·1	5·7	–	5·5	–	**5·0**	–	–	**5·0**
11 °C, 8 h	T	0·0	0·0	1·2	2·4	3·4	4·3	5·2	**6·2**	–	**7·1**	–	–	–	**9·7**
	FI	*12·4*	*12·4*	*12·4*	*12·4*	7·0	6·0	5·7	**5·2**	–	**5·2**	–	–	–	**5·2**
	TS	*14·4*	*14·4*	*14·4*	*14·4*	9·0	8·0	7·7	**8·2**	–	**8·5**	–	–	–	**8·5**
11 °C, 16 h	T	0·0	0·0	1·2	2·4	3·4	4·3	5·2	**6·2**	–	**7·1**	**7·7**	–	–	–
	FI	*12·3*	*12·1*	*12·0*	*12·3*	*12·0*	*11·2*	*11·0*	**6·0**	–	**6·5**	**6·0**	–	–	–
	TS	*14·3*	*14·1*	*14·0*	*14·3*	*14·0*	*13·2*	*13·0*	**8·0**	–	**8·5**	**8·0**	–	–	–
23 °C, 8 h	T	–	0·5	2·3	3·7	5·1	6·4	**7·6**	**8·5**	–	–	–	–	–	**13·4**
	FI	–	*12·1*	*10·9*	8·0	7·5	6·5	**6·5**	**6·5**	–	–	–	–	–	**6·5**
	TS	–	*14·1*	*12·9*	10·0	11·0	10·5	**11·3**	**11·7**	–	–	–	–	–	**12·0**

Values in bold text are for treatments where transfer occurred after FI and bold underlined text are treatments where transfer occurred after TS. Italicized underlined values were estimated as described in the Methods.

**Fig. 3.**
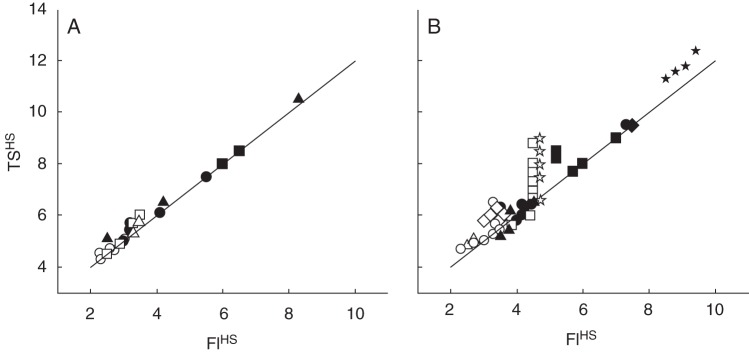
Haun stage at terminal spikelet (TS^HS^) against the Haun stage at floral initiation (FI^HS^) for spring (open symbols) and winter (closed symbols) isolines of ‘Batten’ wheat grown in 16-h Pp (A) and 8-h Pp (B) at 1 (diamonds), 5 (triangles), 8 (circles), 11 (squares) or 23 °C (stars) for different durations before transfer to 23 °C, 16-h Pp conditions. The line represents *y* = 2 + *x*.

To estimate FI^HS^ for treatments where it could not be determined, it was regressed against TS^HS^ for all the treatments where it would be determined (Fig. 3). For the 16-h Pp treatments, there was a strong (*R*^2^ = 0·98) linear relationship with a slope of 1·0 and a *y*-axis intercept of 2·0, suggesting that TS^HS^ – 2·0 would give a suitable estimate of FI^HS^ under these conditions (Fig. 3A). Under 8-h Pp, there were a number of treatments that conformed with the TS^HS^ = FI^HS^ + 2·0 relationship, but there were also a number that deviated from it (Fig. 3B). The non-conforming treatments were those that remained in an 8-h Pp after FI. For example, the spring isoline exposed to 21 d of 23 °C 8-h Pp was moved to 16-h Pp conditions prior to FI and had an FI^HS^ of 4·5 and a TS^HS^ of 6·6 (Table 1). The sub-treatments that received more than 21 d exposure to 23 °C 8-h Pp were moved to 16-h Pp conditions after FI and also had an FI^HS^ of 4·5. However, TS^HS^ increased to 9·0 with 84 d of exposure to 23 °C 8-h Pp. This caused considerable deviation from the TS^HS^ = FI^HS^ + 2 relationship (Fig. 3B). All of the treatments where FI^HS^ could not be fitted experienced 16-h Pp conditions prior to FI^HS^ (Table 2), so we assumed we can safely estimate FI^HS^ for these treatments as TS^HS^ – 2. Thus, from an accurately known FLN we obtained reasonable FI^HS^ and TS^HS^ for all treatments where it could not be quantified from destructive observations (Table 2).

### Apparent gene expression

Molecular assays of gene expression were not conducted because of the technical constraints at the time. We use the evidence presented in the Introduction to assume patterns of gene expression and that the amounts of expression adhere to phenotypic observations to infer apparent expression levels. We assumed arbitrary targets for expression were required to trigger an event and used physiological observations to estimate apparent expression relative to these targets ([Vrn1]_ap_, [Vrn2]_ap_, [Vrn3]_ap_, [Vrn4]_ap_) and the apparent rates of expression relative to these targets (Δ[Vrn1]_ap_, Δ[Vrn2]_ap_, Δ[Vrn3]_ap_, Δ[Vrn4]_ap_).

## ANALYSIS AND RESULTS – ELUCIDATING AND QUANTIFYING RESPONSE MECHANISMS

### Summary of final number of leaves results from Brooking and Jamieson (2002)

**Fig. 4.**
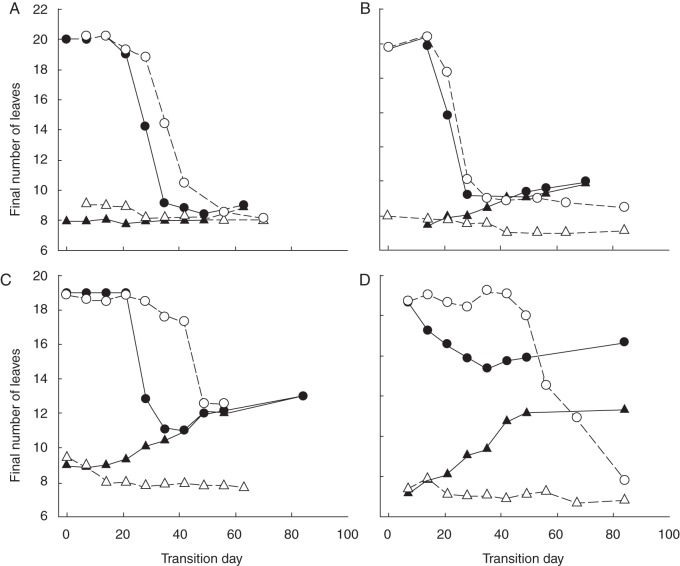
Final number of leaves of spring (triangles) and winter (circles) isolines of ‘Batten’ wheat in response to different durations at lower temperatures and/or 8-h Pp before transfer to 23 °C,16-h Pp conditions. A–C represent temperatures of 5, 8 and 11 °C (respectively) prior to transfer, with filled and open symbols representing 8-h and 16-h Pp (respectively) prior to transfer. (D) Responses for treatments that received 23 °C, 8-h Pp (filled symbols) and 1 °C dark (open symbols) prior to transfer.

Brooking and Jamieson (2002) presented their FLN results in tabular form. However, further insight can be gained by presenting results graphically and comparing isoline and Pp treatments within each temperature treatment (Fig. 4). For all main treatments the sub-treatments that received little or no time in initial treatment conditions (0- and 7-d transfers) showed an FLN of 8–9 for the spring isoline and 19–20 for the winter isoline. In most cases, there were qualitative responses to initial treatment conditions, with longer durations of exposure giving greater increases or decreases in FLN.

For the 5 °C treatment (Fig. 4A), there was little change in FLN for the spring isoline with a small reduction from ∼9·0 to ∼8·5 following 28 d exposure to 5 °C at 16-h Pp and a small increase from ∼8·0 to 9·0 following 49 d exposure to 5 °C at 8-h Pp. The FLN in the winter isoline was ∼20·0 for early transfer treatments and began to decline following 21 d exposure to 5 °C at 8-h Pp. The 5 °C at 16-h Pp treatment took 27 d before a reduction in FLN of the winter isoline was observed. Following 50–60 d of exposure to 5 °C conditions, the FLN of the winter isoline agreed closely with that of the spring isoline in both 8- and 16-h Pp conditions.

In the 8 °C treatment (Fig. 4B), there was also a 1·0 leaf reduction in FLN of the spring isoline under 16-h Pp. However, under 8-h Pp, the FLN increased from 7·5 with 14 d of exposure to 10·1 leaves with 70 d of exposure. For the winter isolines at 8 °C, FLN declined following only 14 d of exposure to vernalizing conditions and reached a minimum of 9·0 leaves with 35 d of exposure. Beyond 35 d of exposure to 8 °C, 8-h Pp, the winter isoline followed the spring isoline, and FLN began to increase again. However, the FLN of the winter isoline remained at 9·0 with longer than 35 d of exposure to 8 °C, 16-h Pp, which was higher than the spring isoline FLN (7·0) for the same exposure.

In the 11 °C, 16-h Pp treatment (Fig. 4C), the FLN for the spring isoline decreased from 9·0 in the 0-d control to 7·8 with longer than 14 d of exposure. Under 11 °C, 8-h Pp conditions there was a steady increase in FLN of the spring isoline up to 13 with 84 d of exposure. The FLN in the winter isoline declined from its maximum of 19·0 following 21 d of exposure to 11 °C, 8-h Pp conditions and following 28 d of exposure to 11 °C, 16-h Pp conditions. Under 11 °C, 8-h Pp the lowest FLN for the winter isoline (11·0) was recorded with 42 d of exposure to vernalizing conditions and followed the same increase in FLN as the spring isoline thereafter. FLN in the 11 °C, 16-h winter isoline treatment did not fall to the same level as the spring isoline under the same conditions and the lowest FLN for this treatment (12·5) was even higher than the lowest FLN in the 8 °C treatment (9·0).

For the 23 °C, 8-h Pp treatment (Fig. 4D), the spring isoline showed a rapid increase in FLN with prolonged exposure to 8-h Pp up to a value of 13·2 with 49 d of exposure. No further increase with additional exposure occurred beyond this. The winter isoline under 23 °C, 8-h Pp conditions showed a reduction in FLN from 18·6 to 15·4 with 35 d of exposure and then an increase in FLN with further exposure to 8-h Pp conditions. For the 1 °C, dark treatments, the spring isoline showed a small reduction in FLN from 9·4 with a 7-d exposure to 8·6 with a 67-d exposure. The winter isoline showed no response until 42 d of exposure to 1 °C, dark conditions but a continued reduction in FLN beyond this to a value of 9·8 with 84 d of exposure.

### When do Pp and vernalization act?

The FLN results of Brooking and Jamieson (2002) show clear quantitative responses and interactions to vernalization and Pp. To quantify these responses, we investigated their effects on FI and TS. First, we considered which phases vernalization affected by looking at the 16-h Pp treatments (Fig. 3A) where Pp was not expected to affect FLN but where 5, 8 or 11 °C temperatures reduced FLN (Fig. 4A–C). Vernalization treatments caused variation in FI^HS^ from 2·5 to 3·5 for spring isolines and 3·0 to 8·0 for winter isolines in those treatments for which it could be defined from primordia counts (Table 2 and Fig. 2). There was a strong correlation between FI^HS^ and TS^HS^, and the regression had a slope of 1·0 and an intercept of 2·0 (Fig. 3A). This shows the duration of the phase from FI to TS was approx. 2·0 HS under 16-h Pp conditions. There were a number of treatments that remained in low temperature conditions well beyond FI^HS^ (Tables 1 and 2) but this did not influence the length of the FI to TS phase. Importantly, this confirms that the vernalization responses were confined to phases prior to FI.

Next we considered the effects of Pp by examining the 8-h Pp treatments (Fig. 3B) where the short Pp induced an increase in FLN (Fig. 4). If we first consider the spring isoline treatments where vernalization had little effect, we can see that the prolonged exposure to 8-h Pp delayed FI^HS^ (Table 1). In some treatments, the relationship between FI^HS^ and TS^HS^ for the 8-h Pp treatments was consistent with the 16-h Pp treatments (Fig. 3B). However, other treatments showed considerable departure from this relationship in an upward direction. Furthermore, all of the data points that deviated from this relationship were treatments that were exposed to 8-h Pp conditions beyond FI (Table 1). This provides strong evidence that short days extended the FI to TS phase and therefore delayed TS.

Finally, we considered situations where Pp and vernalization both influenced development, for example in the winter isoline treatments exposed to an 8-h Pp. Under initial temperature conditions of 5, 8 and 11 °C, the 8-h Pp caused the vernalization response to commence sooner than at the same temperature under 16-h Pp (Fig. 4A–C). This was mirrored by FI^HS^ beginning to decline sooner in the 8-h Pp treatments than in the 16-h Pp treatments (Table 2). The 8-h Pp also gave a reduction in FI^HS^ (Table 2) and FLN (Fig. 4) in the 23 °C treatments relative to the 23 °C 16-h treatments (0-d transfer treatments). In all of these treatments, exposure to 8-h Pp beyond FI^HS^ caused an increase in TS^HS^ (Table 2) and the increase in FLN (Fig. 4) was consistent with the increases shown by spring isolines. This shows that vernalization and Pp interacted to influence the time of FI. Following this, the duration of the FI to TS phase was extended in response to the short Pp.

If we consider these results in the context of assumptions 5–11 (outlined in the Introduction), they are consistent with the notions that:

(1) Both low temperatures prior to FI and spring genotypes enable rapid Δ[Vrn1]. Under high temperatures, spring genotypes will reach the [Vrn1] threshold required for VS, begin expressing Vrn3 and exhibit FI at an earlier HS than winter alleles at the same temperatures.
(2) Long Pp prior to FI upregulates [Vrn2], which increases the [Vrn1] required for VS and delays FI in winter isolines.
(3) Long Pp following VS promotes Δ[Vrn3], which advances the onset of FI and TS.

From this, we can conclude that the model hypothesized in the Introduction is consistent with the physiological observations in response to different environment and genotype treatments. Therefore, we can go on to elucidate and quantify the mechanisms of this model.

### The Pp response

**Fig. 5.**
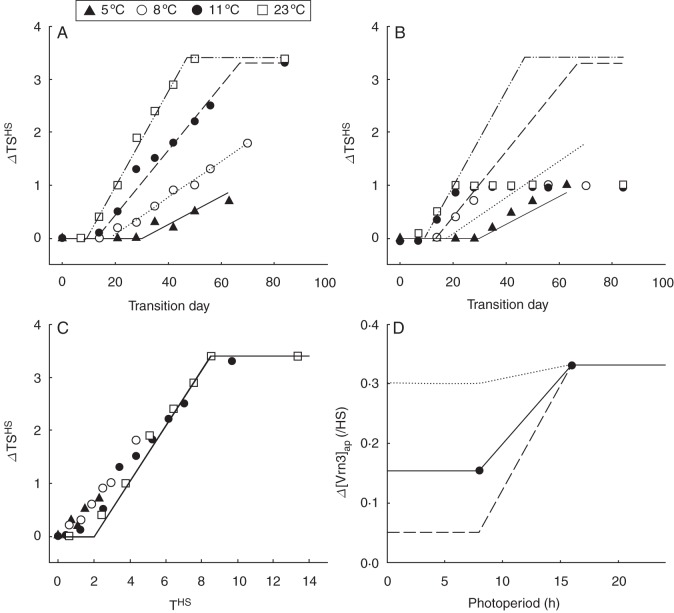
The change in (A) terminal spikelet Haun stage (ΔTS^HS^) and (B) floral initiation Haun stage (ΔFI^HS^) for the spring isoline of ‘Batten’ wheat exposed to 8-h Pp under 5, 8, 11 and 23 °C conditions and then transferred to 23 °C, 16-h Pp conditions at different times following sowing. Lines were fitted by eye to the ΔTS^HS^ data for each temperature in A and the same lines are repeated for reference in B. (C) The change in terminal spikelet Haun stage (ΔTS^HS^) for the spring isoline of ‘Batten’ wheat exposed to 8-h Pp under 5, 8, 11 and 23 °C conditions and then transferred to 23 °C, 16-h photoperiod conditions at different Haun stages (T^HS^). (D) Apparent rates of Vrn3 expression (Δ [Vrn3]_ap_) calculated (circles) for the 23 °C treatments kept at 8- and 16-h photoperiods from emergence to TS. The solid line interpolates between the two points when 8 ≥ Pp ≤ 16 and extrapolates the same value as the points when Pp < 8 or Pp > 16. The other lines demonstrate the expected response of varieties that are more (dash) or less (dotted) Pp sensitive than the spring isoline of ‘Batten’ wheat.

First, we determine a mechanism for quantifying the effects of Pp using the spring isolines where vernalization effects were minor. Figure 4 shows there was a quantitative increase in FLN as spring isolines were exposed to increasing durations of 8-h Pp. The consistent relationship between FLN and TS^HS^ (Jamieson *et al.*, 2007) means this variation can be explained by variation in TS^HS^. Representing these data as an increase in TS^HS^ relative to control treatments (Fig. 5A) shows that, as temperature increased, the rate of increase in TS^HS^ in response to 8-h Pp also increased. For example, 50 d of exposure to 8-h Pp at 5 °C gave a 0·5 increase in TS^HS^ whereas the same period of exposure at 23 °C gave a 3·5 increase in TS^HS^. This supports Assumption 12 (see Introduction) that rates of gene expression are influenced by temperature.

Figure 5A also shows that the response to 8-h Pp saturated at 3·4 HS in the 11 and 23 °C treatments. This can be explained by comparing the timing of transfer to 16-h Pp with TS^HS^ (Table 1). As plants were exposed to longer durations of short Pp, the gap between the HS at transfer and TS^HS^ decreased. No further response occurred in the 11 and 23 °C treatments when they were exposed to 8-h Pp beyond TS^HS^ – 0·5. This suggests that Pp responses stopped and TS was set 0·5 HS prior to the exhibition of TS. TS^HS^ is the sum of the emergence to FI and FI to TS phases. Figure 5B shows the effects of exposure to 8-h Pp on FI^HS^ corresponded to that of the increase in TS^HS^ initially and then the response ceased. This shows that the 8-h Pp extended the duration of the emergence to FI and FI to TS phases in the same way. Thus, it is appropriate to focus solely on the TS data to determine the nature of the temperature and Pp responses.

To quantify the effects of temperature on the magnitude of the Pp-induced increase in TS^HS^, we linked this to vegetative development by plotting the increase in TS^HS^ against the HS of transfer from 8- to 16-h Pp (Fig. 5C). This brought the data closer to a single relationship, but there were still subtle differences. For the 23 °C treatment, TS^HS^ began to increase in response to exposure to 8-h Pp beyond HS 2·0 and showed a linear increase until 8·5 HS exposure, 0·5 HS prior to TS^HS^. The 11 °C treatment was close to this relationship, while the 5 and 8 °C treatments had similar slopes but lower *x*-axis intercepts. If we consider the time of FI and TS to be dependent on adequate [Vrn3] (and subsequent promotion of Δ[Vrn1], as hypothesized in the Introduction), this relationship can be explained as follows: Δ[Vrn3] was dependent on Pp and temperature, and the effects of temperature on Δ[Vrn3] were equivalent to the effects of temperature on HS development. Thus, for a given Pp, the number of HS required to trigger FI and TS will be constant, but the number of days required to trigger FI and TS will depend on temperature. The strong relationship between the increase in TS^HS^ and the HS duration of exposure to 8-h Pp (Fig. 5C) provides evidence that Δ[Vrn3] increases linearly with temperature in the same way that leaf appearance does. Pp responses can therefore be quantified for Δ[Vrn3]_ap_ normalized for the effects of temperature. The differences in the intercepts for the different temperature treatments suggests there was a small vernalization response in the spring isoline with low temperatures inducing the saturation of vernalization and promoting Δ[Vrn3] earlier (see section on low temperature vernalization response below).

We can use these data to calculate the effects of Pp on Δ[Vrn3]_ap_ if we assume an arbitrary threshold of 1·0 to represent the [Vrn3] required to upregulate [Vrn1] enough to cause commitment to TS (Assumption 6 outlined in the Introduction). For the 23 °C treatment, VS occurred and the crop began to respond to Pp conditions after HS 2·0 (the *x*-axis intercept in Fig. 5C) and TS was committed 0·5 HS before it was expressed. In this treatment, TS^HS^ was exhibited at HS 5·5 and 9·0 in treatments exposed to (respectively) 16- and 8-h Pp between HS = 2·0 and TS^HS^ – 0·5 (Table 1). This gives a Δ[Vrn3]_ap_ of 0·333/HS (1/[5·5 – 2·5]) at 16-h Pp and 0·15/HS (1/[9·0 – 2·5]) at 8-h Pp (Fig. 5D). For the same treatments, the exhibition of FI occurred at HS 3·5 and 4·5 when the plants were exposed to (respectively) 16- and 8-h Pp between VS and FI. Assuming the Δ[Vrn3]_ap_ calculated above, this corresponded to [Vrn3] thresholds of 0·33 and 0·31 for the exhibition of FI for the 16- and 8-h treatments, respectively. If we take the average of these values, we can say that a [Vrn3]_ap_ of 0·32 was required to trigger FI.

### Short-day vernalization response

In addition to the Pp response examined above, the winter isoline showed an additional response to 8-h Pp. This was evident for all temperature treatments where FLN started to decrease sooner under 8-h Pp than it did under 16-h Pp (Fig. 4). The most appropriate data for examining this effect are those from the winter isoline grown under initial conditions of 23 °C, 8-h Pp, then moved to 23 °C, 16-h Pp at different times. In this series of treatments, the FLN, FI^HS^ and TS^HS^ all decreased with extended exposure to 8-h Pp up to 35 d and then TS^HS^ and FLN began to increase again in response to additional exposure to 8-h Pp (Fig. 4; Table 2). The increase in FLN and TS^HS^ beyond 35 d of exposure was probably due to Pp promoting Δ[Vrn3] _ap_ as discussed above. However, the reduction in FLN prior to this is due to short-day vernalization.

**Fig. 6.**
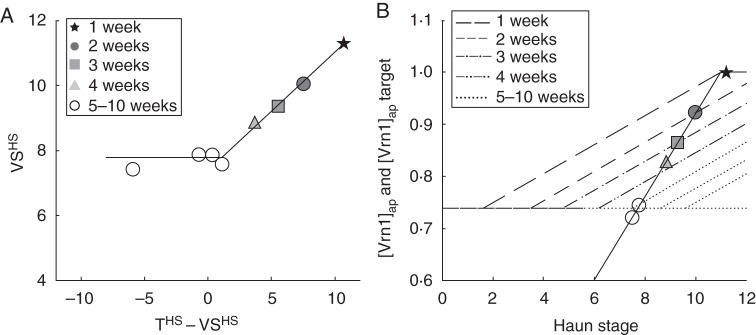
(A) The Haun stage at which vernalization saturation occurred (VS^HS^) against the number of Haun stages of 16-h Pp that were experienced prior to VS (T^HS^ – VS^HS^) for the winter isoline of ‘Batten’ wheat grown under initial conditions of 23 °C and 8-h Pp for 1, 2, 3, 4 and 5–10 weeks before transfer to final control conditions (23 °C, 16-h Pp). (B) The apparent expression of Vrn1 ([Vrn1]_ap_, solid line) for the winter isoline of ‘Batten’ wheat grown at 23 °C, 16-h Pp compared with vernalization targets (broken lines) for 1, 2, 3, 4 and 5–10 week transfer treatments. Symbols mark the [*Vrn1*]_ap_ that would have occurred at VS^HS^ displayed in A. Vernalization target is 0·74 + [Vrn2]_ap_, which increases by 0·028/HS following a 1·1 HS lag after transfer to 16-h Pp conditions.

To explain short-day vernalization responses, it was necessary to separate the effects of Pp on the time of VS (when Δ[Vrn3]_ap_ becomes positive) from the effects of Pp on Δ[Vrn3]_ap_ and the time of FI. The Pp response mechanism outlined above can be used to back-calculate the HS at which VS occurred (VS^HS^) from values of FI^HS^ using: (1) the Pp conditions that were encountered prior to FI, (2) the Δ[Vrn3] _ap_ in response to that Pp and (3) the [Vrn3] target of 0·32 to pass from VS to FI. These calculations were made in a spreadsheet and results are displayed for the 23 °C, 8-h winter isoline treatments in Fig. 6A. The assumptions (from the Introduction) that are important to the interpretation of these data are:

Assumption 9, that VS occurs when [Vrn1] is sufficient to repress [Vrn2] and promote Δ[Vrn3], and
Assumption 10, that Δ[Vrn2] increases with longer Pp.

Dubcovsky *et al.* (2006*a*) have shown that [Vrn2] was undetectable under 8-h Pp but was high under 16-h Pp. This implies that the [Vrn1]_ap_ required to achieve VS (the [Vrn1] target) will be lowest at emergence, remain low under 8-h Pp conditions but will increase with exposure to 16-h Pp. To investigate this we plotted VS^HS^ against the HS duration of exposure to 16-h Pp prior to VS (T^HS^ – VS^HS^, Fig. 6A). When T^HS^ – VS^HS^ was less than 1·1, plants only encountered short Pp up to 1·1 HS before VS. In these cases, the VS^HS^ was constant at 7·8. As treatments were exposed to longer durations of long Pp prior to VS^HS^ – 1·1, there was a quantitative increase in VS^HS^ up to a value of 11·2 for control treatments that were exposed to 16-h Pp until or beyond HS 10·4. This suggests that there was a 1·1 HS lag period prior to the onset of positive Δ[Vrn2]_ap_ under 16-h Pp. This was followed by an increase in the [Vrn1] target and the subsequent VS^HS^ with exposure to 16-h Pp beyond HS 1·1.

To quantify this response, we used the control treatments that were in 16-h Pp for their entire duration. Assuming that [Vrn2]_ap_ was zero until HS 1·1 and then shows a constant accumulation to a relative value of 1·0 and VS (HS 10·4), this gives a relative Vrn2 expression rate at 16-h Pp of 1/(10·4 – 1·1) = 0·107/HS. To determine the effects that [Vrn2]_ap_ has on the [Vrn1] target needed to promote Δ[*Vrn3*] we need to consider the data in Fig. 6A with regard to [Vrn1]_ap_. These treatments were all grown at 23 °C, so Δ[Vrn1]_ap_ would have been at the background rate (quantified in the next section). Background [Vrn1]_ap_ was plotted against HS in Fig. 6B with symbols marking the position of VS^HS^ on this curve for each of the 23 °C 8-h treatments. All treatments grown under 8-h Pp until VS had a VS^HS^ of ∼7·8. At this HS, [Vrn1]_ap_ had accumulated to a value of 0·74 (Fig. 6B). This suggests that in the absence of [Vrn2], the [Vrn1] target to trigger VS was 0·74. Assuming the maximum [Vrn1]_ap_ was 1·0, then exposure to 16-h Pp from emergence to VS gave an increase in the Vrn1 target of 0·26. Assuming a constant 0·107/HS Δ[Vrn2]_ap_ (calculated above) from 1·1 HS after emergence, then the effect of 16-h Pp on the [Vrn1]_ap_ target was an increase of 0·028/HS. To check the assumption of a linear increase, we plotted a linear increase in the [Vrn1]_ap_ target from an initial value of 0·74 with an increase of 0·028/HS from 1·1 HS after the treatment was moved from 8- to 16-h Pp (Fig. 6B). These Vrn1 targets intercept the base [Vrn1]_ap_ at an HS that agreed closely with the observed VS^HS^ for the corresponding treatments (Fig. 6B).

### Low-temperature vernalization response

**Fig. 7.**
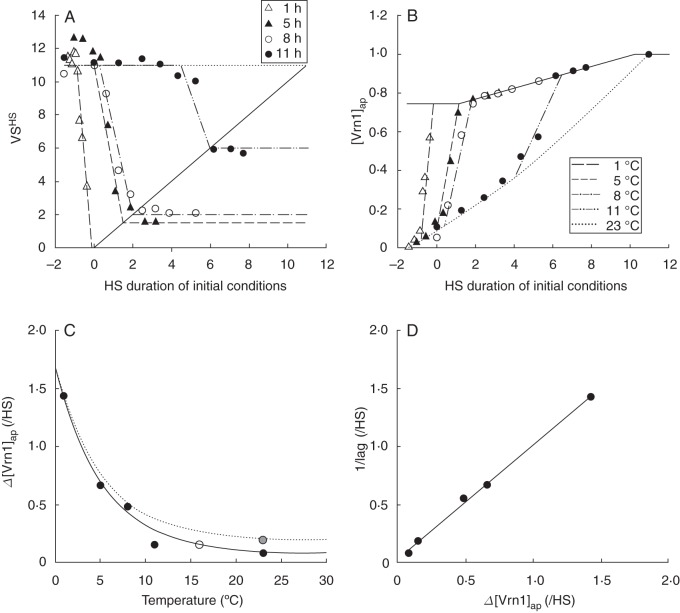
(A) The Haun stage at which vernalization saturation occurred (VS^HS^) against the Haun stage (HS) duration of exposure to initial treatment conditions of 1, 5, 8 and 11 °C temperature and 16-h photoperiod conditions prior to transfer to 23 °C, 16-h photoperiod conditions for the winter isoline of ‘Batten’ wheat. The solid line marks *y* = *x* and the broken lines represent the reduction in VS^HS^ for 1, 5, 8, 11 and 23 °C. (B) Apparent Vrn1 expression rates (Δ[*Vrn1*]_ap_), estimated from data in A and the estimated [Vrn1] target (solid line). Lines fitted by eye have a lag phase where the increase in Δ[Vrn1] was at the base rate and a 2nd phase where Δ[Vrn1] increased. (C) The Δ[Vrn1]_ap_ during the 2nd phase from B against temperature. The open symbol plots the value for the 11 °C treatment at a temperature of 16 °C; the grey symbol is Δ[Vrn1]_ap_ for the spring isoline grown in 23 °C, 16-h photoperiod conditions. Regression (solid line) is of the form *y* = 0·08 +1·6e^−0·19^^*x*^ (*R*^2^ = 0·98) and the dotted line represents the expected response of a less vernalization-sensitive genotype. (D) The reciprocal of the HS duration of the lag period (1/lag) against the Δ[Vrn1]_ap_ during the 2nd phase for the corresponding temperature treatment. Regression (solid line) is of form *y* = 0·03 + 0·98*x* (*R*^2^ = 0·99).

To elucidate a mechanism for quantifying the effects of low-temperature vernalization, we use the winter ‘Batten’ isoline grown under 16-h Pp. This ensures Pp responses will be the same before and after transfers to 23 °C conditions. To analyse vernalization responses, we have plotted the VS^HS^ (calculated as described in the previous section) against the duration of exposure to initial treatments where duration was quantified by the HS at which the treatments were moved from initial to control conditions (Fig. 7A). First, we discuss the extremes of the data to establish the boundaries for response. A problem encountered when analysing vernalization in relation to HS was that the scale begins at emergence, but wheat responds to vernalization from imbibition. For example, all the 1 °C treatments were moved to 23 °C, 16-h Pp conditions prior to HS 0 (Table 2), but the treatments that encountered more than 49 d of 1 °C temperatures showed a reduction in FLN (Fig. 4). To account for this, we extended the HS into negative values with a value of –1·5 used to represent the HS at germination, which was assumed to closely follow imbibition on the day treatments were established. The value of –1·5 was calculated from the Tt from sowing to emergence (110 °Cd) and assuming ‘phyllochron’ was 60 % of the base phyllochron (120 °Cd) for negative HS. The maximum VS^HS^ (maxVS^HS^) was ∼11·0 with the controls that encountered 23 °C, 16-h Pp conditions from HS – 1·5 until VS and for other treatments that were ineffective at inducing a reduction in VS^HS^. The maxVS^HS^ represents the latest possible HS at which VS will occur when no induction of vernalization response is encountered. VS can occur prior to emergence (HS ≤ 0) but subsequent developmental processes begin at HS 0·0 (requiring Pp perception), so it was suitable to assume a minimum VS^HS^ (minVS^HS^) of zero for these analyses. The *y* = *x* line connects minimum and maximum VS^HS^ values and represents a threshold below which no further vernalization response was possible (i.e. encountering cool conditions beyond a particular HS cannot cause VS to occur earlier than that HS).

Having established the response boundaries in Fig. 7A, we next consider the pattern within. For all temperature treatments, there were three clear phases of response to increased exposure of initial treatment conditions that need to be considered. These were: (1) a lag phase where reduction in VS^HS^ was not evident followed by, (2) a response phase where VS^HS^ declined and (3) a final phase where no further response occurred. The 1 °C treatment was the most effective for vernalization, passing from the lag phase to the reduction phase earliest, having the fastest reduction and reaching the final non-response phase earlist (Fig. 7A). As initial treatment temperatures increased, the HS of transition from the lag phase became later and the rate of decline in VS^HS^ in the response phase decreased. As a result, the HS timing of transition to the final phase (the *x* value of the response line when it intercepts the *x* = *y* threshold) became later, and the lowest value of VS^HS^ (the *y* value of the response line when it intercepts the *x* = *y* threshold) increased with higher temperature treatments.

To interpret the responses shown in Fig. 7A, it is useful to consider the timing of VS as the accumulation of sufficient [Vrn1] to repress Δ[Vrn2] and promote Δ[Vrn3] (Assumption 9 from the Introduction). If we first consider the controls where no vernalizing conditions were encountered, VS^HS^ was 11·0. The interpretation of this is that there is small positive background Δ[Vrn1] occurring in any facultative cereal, so the cereal will saturate its vernalization response eventually, even in non-vernalizing conditions. If cereal genotypes displayed obligate vernalization behaviour, this would imply background Δ[Vrn1] is zero. Assuming a constant Δ[Vrn1]_ap_/HS from imbibition (HS – 1·5) until VS (HS 11·0) under non-vernalizing conditions and assuming a [Vrn1]_ap_ target of 1·0 at VS, the base rate of Δ[Vrn1]_ap_ was 1/(11 + 1·5) = 0·08/HS. For the spring isoline, VS^HS^ was 2·5 in 23 °C, 16-h Pp conditions, which means the base rate of Δ[Vrn1]_ap_ was 1/(2·5 + 1·5) × 0·76 = 0·19/HS. If we consider the winter isoline treatments that were exposed to vernalizing temperatures, in cases where exposure to vernalizing conditions was long enough, VS occurred earlier than 11·0 (Fig. 7A). Therefore, additional to the base rate of Δ[Vrn1]_ap_, there was also an induced increase in Δ[Vrn1]_ap_ that occurred in response to low temperature.

The effect of temperature promoting an increase in Δ[Vrn1]_ap_ was represented for each treatment by dividing its VS^HS^ value by maxVS^HS^. The maximum of this ratio and the [Vrn1]_ap_ resulting from base expression for the corresponding HS at which exposure to vernalizing conditions stopped was multiplied by the [Vrn1]_ap_ target (described in the previous section) at that HS. The resultant data are displayed in Fig. 7B. These data can be interpreted as representing a three-phase response. In the first phase, the increase in [Vrn1]_ap_ was equal to the base Δ[Vrn1]_ap_. This rate increases in the second phase and is zero in the third phase. The two parameters from this model that are important for describing low-temperature vernalization responses are the slope of the relationship during the second phase and the HS duration of the first phase. The slope of the second phase represents the Δ[Vrn1]_ap_ promoted by low temperature. This was plotted against the corresponding temperature for each treatment (Fig. 7C) and showed an exponential reduction from a value of 1·4/HS at 1 °C to the base value of 0·08/HS at 23 °C. The lag phase can be interpreted as some factor repressing Δ[Vrn1]_ap_ that must be downregulated before the cold-induced promotion of Δ[Vrn1]_ap_ can occur. The reciprocal of the lag duration was taken to represent the rate of progress through the lag phase. This was proportional to Δ[Vrn1]_ap_ of the second phase (Fig. 7D), which suggests the down-regulation of the factor that is initially repressing the cold-induced promotion of Δ[Vrn1]_ap_ responds to temperature in the same way as the promotion of Δ[Vrn1]_ap_. For each treatment, there were a number of sub-treatments that experienced some vernalizing conditions but did not show a reduction in VS^HS^ (Fig. 7A). If the factor that was initially repressing of cold-induced promotion of Δ[Vrn1]_ap_ was gradually downregulated following exposure to cold, we would expect some reduction, but this was not the case. Thus, the lag period suggests that this repressor was rapidly upregulated again upon exposure to 23 °C. The interpretation for sub-treatments that were not exposed to vernalizing conditions for long enough for this promoter to be downregulated sufficiently to allow Δ[Vrn1]_ap_ within the cool treatment phase was that the repressor was quickly upregulated again on exposure to high temperatures, Δ[Vrn1]_ap_ remained at the base rate and VS^HS^ occurred at its maximum value.

## DISCUSSION – A MODEL THAT INTEGRATES PHYSIOLOGICAL AND MOLECULAR BIOLOGICAL CONCEPTS

**Fig. 8.**
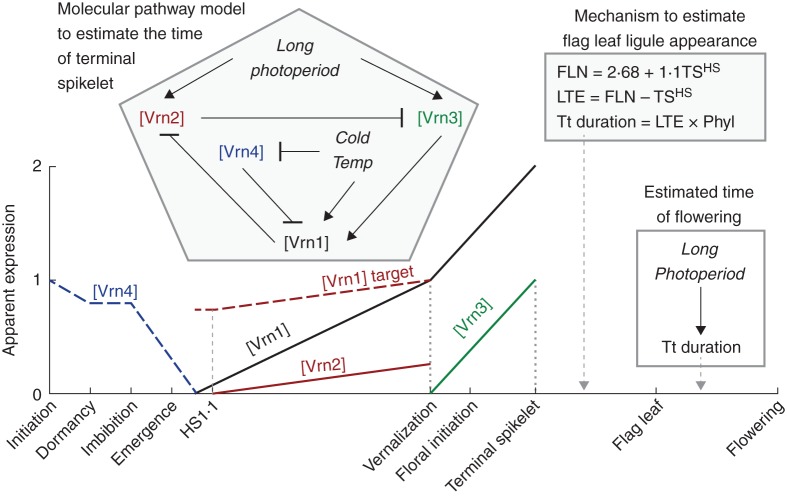
Schematic representation of the integrated model. The crop must pass through each of the phases along the *x*-axis to reach anthesis. Temperature per se controls the progression through each phase in combination with the factors presented. Temperature and photoperiod control the expression of *Vrn1*, *Vrn2*, *Vrn3* and *Vrn4* genes as demonstrated by the scheme within the pentagon (pointed arrows show promotion and flat arrows show repression) and subsequent amount of [Vrn1], [Vrn2], [Vrn3] and [Vrn4] protein expressed as demonstrated by the lines on the graph. The amount of these proteins controls the timing of vernalization and terminal spikelet.

The aim of this paper was to elucidate and quantify a model that integrates molecular biology and plant physiology to provide a framework to link anthesis time genotype to quantitative predictions of time of anthesis in different environments. This was achieved by a phase-based physiological model to link progression through particular phases to the extent of gene expression (Fig. 8). The first step in development of such an approach was to determine a phenological model that provides a suitable structure for integrating molecular models (i.e. to determine the key stages on the *x*-axis of Fig. 8).

### A phase-based model

All physiological wheat anthesis models use phases between observable stage events to simulate progress to anthesis; they differ in which stages they use as phenological markers. ARCWHEAT1 (Weir *et al.*, 1984) uses imbibition, emergence, FI, DR, TS and anthesis. SIRIUS uses the same stages to emergence, followed by FL and anthesis stages. However, the necessary phases need to be reconsidered to link explicitly to gene expression drivers. Assumptions 1–4 (from the Introduction) infer the need for TS, FL and anthesis (Fig. 8). To allow more detailed treatment of the controls of vernalization and Pp, it is necessary to separate the duration from sowing to TS into two phases: (1) a vegetative phase when the crop responds to both cold and photoperiod until VS, and (2) a reproductive phase beyond VS in which the crop responds to photoperiod alone. The ARCWHEAT model uses the DR stage to separate vegetative and reproductive phases. However, Hay and Ellis (1998) have cautioned against the use of this stage because it does not relate well to the transition to reproductive development. Other authors (Kirby, 1990; Brooking, 1996; Brooking and Jamieson, 2002) suggested the onset of an increase in primordium initiation to represent the transition from vegetative to reproductive growth. We tested this by determining FI^HS^ and TS^HS^ (Fig. 2; Table 2) for a wide range of treatments and analysing the effects of vernalization and Pp on the timing of these stages. Results showed (Fig. 3) that vernalization only affected FI^HS^, whereas photoperiod affected both FI^HS^ and TS^HS^. DR occurs some time after the increase in primordia initiation, which means it is an inaccurate indicator of the time of VS.

While the effects of vernalization are all set prior to FI, there was also a small Pp effect on the timing of this event that was equivalent to the Pp response of TS (Fig. 5B). We interpreted this as the plant requiring 0·3 [Vrn3]_ap_ for FI to occur. Thus, FI represents a morphological change (in primordium initiation rate) rather than a physiological change (when the crop stops responding to vernalization) and is not a useful stage to represent in a model. We have defined a stage called VS which occurs prior to FI and represents the transition from vernalization to Pp response. While this stage is useful from a modelling perspective, it may prove problematic from an experimental perspective as it is not readily observable and in this study it required detailed experimental structure and analysis to calculate it *a posteriori*. Measurement of gene expression could provide a method for determination of the timing of VS experimentally with the expression of *Vrn3* occurring following this event.

Additional stages are also needed before VS as there are phases when distinct physiological behaviour is displayed. The first phase to consider is between initiation of an embryo and when it becomes dormant while still on the mother plant. Cool conditions can induce a vernalization response during this phase that is ‘remembered’ when the seed imbibes and begins autonomous growth (Gregory and Purvis, 1936). This is followed by a dormant phase between harvest and sowing when no physiological response occurs. These stages can be included in a hypothetical model (Fig. 8) for completeness, but in most cases the extent of vernalization during grain development would be nil so these phases could be omitted. The next phase begins when the seed imbibes (Fig. 8), and the plant is able to perceive and respond to vernalization again (Flood and Halloran, 1986). This is followed by emergence, at which point the plant can also perceive and respond to Pp (Cooper, 1956).

### Integrating concepts from molecular models for vernalization and Pp responses

The linkage between physiological and molecular models comes with the assumption that the occurrence of a phenological stage is triggered by the accumulation of adequate levels of gene expression (Assumption 5, from the Introduction). An example of how expression profiles would change as the crop develops is given in Fig. 8. We took the model presented by Li *et al.* (2011) as the basis for how environment and genes interact to control the expression signals needed to trigger phase changes (Assumptions 6–9 in the Introduction and the contents of the pentagon in Fig. 8) and then analysed physiological observations to infer environmental responses of apparent gene expression. In this context, we were able to explain all of the physiological observations of an experiment that presented a wide range of vernalization and Pp treatments for two genotypes. This provides encouraging signs that molecular and physiological studies can be reconciled by linking phenological stage changes to underlying gene expression. We note that there is not complete agreement in the structure of the molecular model for flowering, with Chen and Dubcovsky (2012) presenting an alternative. We have not presented full results here but the current model can be easily reformulated to explain the phenological observations of Brooking and Jamieson (2002) with Vrn3 upstream of Vrn1.

#### Temperature

Temperature per se is one of the most important controllers of anthesis time. This is to be expected as temperature has a strong influence on the rate of chemical reactions that underlie biological processes. The effects of temperature are captured in most models by using Tt to drive the rate of developmental progress through phases. However, temperature per se can also influence the extent of Pp and vernalization responses that determine the amount of Tt that must be accumulated to complete a phase. For instance, Fig. 5A shows an increase in temperature increased the effect that exposure to 8-h Pp had on the timing of TS. Yan and Wallace (1996) have also presented a range of data showing the effects of temperature on Pp response of anthesis time in a range of crops. They considered this to be due to the effects of temperature on the expression of Pp genes. They presented an empirical model for quantifying these effects. Leaf appearance is a reliable measure of the temperature per se and HS can be accurately estimated from the correct temperature data (Fig. 1) (Jamieson *et al.*, 1995). Therefore, we used this as a metric to account for the effects of temperature per se. Expressing change in the timing of TS as a function of HS exposure to short Pp, we were able to produce a unifying relationship that explained the response of TS to short Pp across a range of temperatures (Fig. 5C). From this, we assumed that the expression rate of all genes should be represented as a function of HS development to account for the effects of temperature per se.

#### Vernalization target

The model we propose has VS as an explicit stage (Fig. 8) that the crop will achieve once [Vrn1] is sufficient to repress Δ[Vrn2] and promote Δ[Vrn3] (Assumption 9, Introduction). To quantify when this happens we need to know the [Vrn1] target and this was quantified as 0·76 under continuous 8-h conditions (Fig. 6). Dubcovsky *et al.* (2006*a*) showed that [Vrn2] was nil at a Pp of 8 h but expressed at high levels at 16-h Pp. This suggests that the target value of 0·76 represents the [Vrn1] required to promote Δ[Vrn3]. The [Vrn1] target increased linearly at 0·028/HS when exposed to 16-h Pp for more than 1·1 HS. This is consistent with the upregulation of [Vrn2] in response to long Pp (Dubcovsky *et al.*, 2006*b*; Trevaskis *et al.*, 2006; Sasani *et al.*, 2009) and the observation that the presence of a *Vrn2* gene only gave differences in anthesis time under long day conditions (Karsai *et al.*, 2005). This suggests that Δ[Vrn2] is promoted by long Pp and the higher [Vrn2] is, the more Vrn1 is required to repress its effects (allowing Vrn3 to be expressed) and so the higher the [Vrn1] target for VS will be (Fig. 8). Relating the vernalization target to [Vrn2] and having this increase in response to long Pp provides a novel mechanism for quantifying short-day vernalization responses (Brooking and Jamieson, 2002; Allard *et al.*, 2012) and a link for the effects of *Vrn2* genetic variation into a quantitative anthesis time model. Unfortunately, we have only two Pp treatments in the dataset analysed and are unaware of other datasets where frequent measurements of HS and primordium number (to allow the same analysis) have been conducted with other photoperiods. To establish a base on which further progress can be made, we assume Δ[Vrn2] will be zero at 8-h Pp, a linear increase in Δ[Vrn2] up to 0·028/HS at a 16-h Pp and no response thereafter.

#### Vernalization response

With the effects of Pp on vernalization quantified it was also necessary to quantify the effects of temperature on Δ[Vrn1] to determine when the target is reached and VS occurs (Fig. 8). We analysed VS relative to HS exposure to low temperatures (Fig. 7A) and calculated [Vrn1]_ap_ from this (Fig. 7B). The result was an exponential temperature response for vernalization where Δ[Vrn1]_ap_/HS was highest at 1 °C and decreased to a low base level at 23 °C (Fig. 7C). This is consistent with the upregulation of [Vrn1] that occurs under lower temperatures (Trevaskis *et al.*, 2007; Diaz *et al.*, 2012). However, this response differs from the vernalization response presented by Brooking (1996), which increased from a low rate at 0 °C to a maximum at 8 °C and then decreased to have no vernalization at temperatures greater than 16 °C. The reason for the different response is the representation of vernalization rates (Δ[Vrn1]_ap_) relative to HS in the current approach. The vernalization response presented by Brooking (1996) differed from that of previous authors because he took explicit account of the confounding effect that vegetative development during vernalization has on FLN. However, Brooking (1996) calculated vernalization rates in chronological time rather than developmental time and thus maintained temperature effects per se as a confounding factor on the expression of the vernalization signal, [Vrn1]. By calculating Δ[Vrn1]_ap_ relative to HS, the presented model removes the confounding effects of temperature per se on both vegetative development and Δ[Vrn1]_ap_.

The vernalization response in Fig. 7C contains a low base Δ[Vrn1]_ap_ at high temperatures for the winter ‘Batten’ isoline. This reflects the fact that the winter isoline did eventually flower even when kept at 23 °C from sowing onward. This is the case for most genotypes of winter wheat, which express a facultative rather than an obligate vernalization response (Davidson *et al.*, 1985). This base rate suggests that even in winter isolines, the *Vrn1* gene is active at high temperatures. Variation in the vernalization sensitivity among genotypes is associated with changes in the ability of *Vrn1* alleles to bind with repressors (Trevaskis, 2010) or the copy number of *Vrn1* genes affecting rates of [Vrn1] increase (Diaz *et al.*, 2012). Presumably the temperature response in Δ[Vrn1]_ap_ shown in Fig. 7C is also caused by a reduction in the activity of the *Vrn1* repressor as temperatures decline and this allows an increase in Δ[Vrn1]_ap_. Alternatively, it may be the chromatin state of the *Vrn1* gene that responds to temperature rather than a repressor (Oliver *et al.*, 2013). The positive value of base Δ[Vrn1]_ap_ must mean that even in winter alleles, down-regulation is still not completely effective. All this suggests that the effects of the *Vrn1* genotype may be quantified within the proposed model (Fig. 8) by relating the *Vrn1* alleles of a genotype to the base Δ[Vrn1]_ap_ expression at high temperatures. So the expected vernalization response of a spring type could be represented by the dotted line in Fig. 7C and intermediate sensitivities would be somewhere in between. There was evidence of a small vernalization response in the spring isoline of ‘Batten’ wheat, with the timing of FI varying by about 1·0 HS between temperature treatments (Fig. 6D; Table 1).

In addition to quantifying the effect of accumulated cold on acceleration of VS (and inferred effects on Δ[Vrn1]), there was also a clear lag period where short exposure to low temperatures did not reduce VS^HS^ (Fig. 7A). This suggests there is another repressor that must be downregulated to enable promotion of Δ[Vrn1] (Fig. 8). The *Vrn4* gene may play a role here, as Yoshida *et al.* (2010) has shown the *Vrn4* loci to influence the lag period of vernalization. In our proposed model we speculate that Vrn4 represses Vrn1 (blocking cold-induced promotion of Vrn1) and must be repressed to allow promotion of Δ[Vrn1] above the base rate. The reciprocal of the lag duration was directly proportional to Δ[Vrn1]_ap_, suggesting the downregulation of [Vrn4] has the same temperature sensitivity as the promotion of Δ[Vrn1] over the range of temperatures tested (Fig. 7D). Thus, the proposed model starts with [Vrn4]_ap_ at a value of 1·0 and its levels are decreased in response to temperature at the same rate that [Vrn1] is increased in response to temperature (Fig. 7C). If the crop is exposed to temperatures of 23 °C, [Vrn4] increases quickly again so no promotion of Δ[Vrn1] occurs if the duration of cold exposure is insufficient for [Vrn4] to reach 0. Other authors (Wang *et al.*, 1995*a*, *b*) have shown genetic variation in the duration of this lag period, suggesting it is under genetic control. Possibly, genotypes missing the *Vrn4* gene will not exhibit a vernalization response lag (Yoshida *et al.*, 2010) whereas those with different alleles or copy numbers of this gene may exhibit differing lengths of lag period. Further work is required to confirm the role of *Vrn4* and quantify the relationship between genetic variation in *Vrn4* and the parameters of this vernalization response model.

#### Pp responses

There was a Pp response in the vernalization process which was evident because short Pp reduced FLN. Following vernalization the crop showed the opposite response where short Pp increased FLN (Figs 4 and 5). The hypothetical model (Fig. 8) assumes that a [Vrn1]_ap_ of 2·0 is required to trigger TS but that [Vrn1]_ap_ will only reach a value of 1·0 in the absence of Vrn3. We assume a parallel increase in [Vrn1] in response to [Vrn3], so while it is Vrn1 that controls the reproductive response (Assumption 5), the effects of Pp on the timing of TS can be interpreted in terms of the expression of Vrn3.

The duration of the early reproductive phase was shortest with constant exposure to 16-h Pp and showed a quantitative increase with response to increasing periods of exposure to 8-h Pp. This is consistent with studies showing Δ[Vrn3] increases in response to long Pp and the notion that a certain [Vrn3] (and subsequently [Vrn1]) must accumulate to trigger TS (Assumption 5 in the Introduction, Fig. 8). Although the experiment of Brooking and Jamieson (2002) contained a number of treatments, there were only two Pp levels and we are not aware of other datasets where frequent measurements of HS and primordium number (to allow the same analysis) have been conducted with other Pp. Flag leaf number in response to Pp shows a linear (Brooking *et al.*, 1995) increase in response to increasing Pp. To establish a base for the hypothetical model on which further progress can be made, we assume that a linear interpolation of Δ[Vrn3] in response to Pp (Fig. 5D) but acknowledge that further research is required to verify this Pp response relationship.

### After terminal spikelet

The duration of the stem extension phase is determined by Tt accumulation, the phyllochron of the crop and the number of leaves remaining to emerge (LTE). Figure 8 shows the scheme that is used for calculating LTE, which is dependent on TS^HS^ and its effects on FLN (Jamieson *et al.*, 2007). Other authors have shown small vernalization responses in the phase from the end of the vegetative phase until anthesis (Slafer and Rawson, 1994; Gonzalez *et al.*, 2002). This can be explained by the effect vernalization has on TS^HS^ and its subsequent effect on LTE (Fig. 8). A treatment that gives more effective vernalization will have a lower VS^HS^ (and subsequent TS^HS^) and so have fewer leaves to appear following TS. This vernalization treatment will result in a shorter duration from TS to anthesis although the response was set prior to VS. The length of the ear development phase has also been demonstrated to have considerable Pp sensitivity (Slafer and Rawson, 1996; Gonzalez *et al.*, 2002; Brown *et al.*, 2012). We have not attempted to quantify this response in the current paper but this response will be needed in the implementation of a model to predict anthesis time. Similarly, we are unaware of any molecular studies that look to isolate the role of particular genes in the control of Pp response during this specific phase. Therefore, further research will be required to identify the controlling genes and quantify the effects of allelic variation on the nature of the Pp response to be able to construct a model that effectively enables quantitative estimations of anthesis time from genotype and environment information.

## FORMAL MODEL DESCRIPTION AND APPLICATION

### Model description of environmental responses

The final section of this paper provides a description of the proposed model (Fig. 8) and some hypotheses about how the model parameters would relate to genotype. The model contains the following phases: 1, embryo development → 2, dormant → 3, imbibed/emerging → 4, vegetative → 5, early reproductive → 6, pseudo-stem extension → 7, ear development. The bounds of these phases are marked by the stages embryo initiation, dormancy, imbibition, emergence, VS, TS, FL and anthesis (Fig. 8).

Vegetative development is the group of phases where vernalization occurs (Phases 1, 3 and 4) and FLN shows a reduction in response to cold and an increase in response to long Pp. Vernalization refers to the expression of sufficient [Vrn1] to enable the promotion of Δ[Vrn3]. [Vrn4] represses Δ[Vrn1]. It begins at a value of 1·0 for ‘Batten’ wheat and is calculated daily in response to mean temperature (*T*) as:
(1)
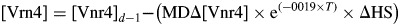

where [Vrn4]_*d* − 1_ is the expression of [Vrn4] from the previous day, Δ[Vrn4] is the rate of change in [Vrn4] with respect to the rate of change in HS and MDΔ[Vrn4] is a parameter representing the maximum rate of downregulation of [Vrn4] and is measured by the Δ[Vrn4] at 1 °C. This had a value of 1·6/HS for winter ‘Batten’. If the crop is exposed to cool conditions, [Vrn4] (Fig. 8) falls quickly and may reach 0 prior to emergence. However, under warm conditions, it falls slowly, delaying the onset of Δ[Vrn1] responses to temperature. If the crop is exposed to 23 °C, [Vrn4] is returned to 1·0. [Vrn1] is calculated daily as:
(2)
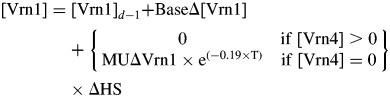

where Δ[Vrn1] is rate of change in [Vrn1] with respect to rate of change in HS, BaseΔ[Vrn1] is a parameter representing Δ[Vrn1] that occurs regardless of temperature and MUΔ[Vrn1] is a parameter representing the maximum rate of upregulation of [Vrn1] which is the rate measured at 1 °C. MUΔ[Vrn1] had a value of 1·52/HS for ‘Batten’ wheat and BaseΔ[Vrn1] was 0·08/HS for the winter ‘Batten’ isoline. Under cool conditions, [Vrn1] (Fig. 8) would increase quickly but under warm conditions the increase would be slowed. The [Vrn1] required to promote Δ[Vrn3] depends on photoperiod.

The upregulation of [Vrn3] occurs when vernalization saturation has occurred. This requires [Vrn1] to reach a value of 0·76 and this may happen before or after emergence. If it happens before emergence, the crop will pass directly from Phase 3 to Phase 5. If it happens after emergence, the crop will have a vegetative phase (Phase 4) where it is emerged but still requires vernalization. During Phase 4, Vrn2 may be expressed and this would repress Δ[Vrn3]. Vrn1 in turn represses Δ[Vrn2] but more [Vrn1] is required to achieve this. Thus, Vrn2 expression has the effect of increasing the target for [Vrn1] to achieve vernalization (when FLN stops responding to cold and shows a decrease in response to long Pp). During Phase 4 Vrn2 is expressed in response to Pp as:
(3)
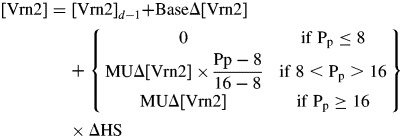

where BaseΔ[Vrn2] and MUΔ[Vrn2] are rates of change of [Vrn2] with respect to rate of change in HS. BaseΔ[Vrn2] had a value of 0/HS and MUΔ[Vrn2] had a value of 0·026/HS for ‘Batten’ wheat. The target level of [Vrn1] is calculated as 0·76 + [Vrn2]. Under Pp of ≤8 h, [Vrn2] (Fig. 8) would remain at zero and the [Vrn1] target would remain at 0·76. At Pp > 8 h, [Vrn2] and the [Vrn1] target would increase. Vernalization occurs and the crop passes to Phase 5 (early reproductive) when [Vrn1] ≥ this target.

Once [Vrn1] has reached the target level, the plant is vernalized and the effects of photoperiod on Δ[Vrn3] will be evident. The [Vrn3] is calculated in Phase 5 in response to Pp as:
(4)
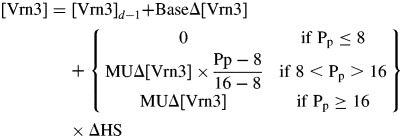

where BaseΔ[Vrn3] and MUΔ[Vrn3] are rates of change of [Vrn3] with respect to rate of change in HS. BaseΔ[Vrn3] had a value of 0·15/HS and MUΔ[Vrn3] had a value of 0·18/HS for both isolines of ‘Batten’ wheat. [Vrn3] upregulates [Vrn1] in parallel and we assume that when [Vrn3] reaches a value of 1·0, [Vrn1] will be promoted to sufficient levels for the plant to commit to reproductive growth and terminal spikelet will be visible 0·5 HS later. Under short Pp, [Vrn3] (Fig. 8) would be slowly upregulated but under longer Pp it would increase rapidly to accelerate progress toward TS.

At TS the fate of all other primordia on the apex is also set. The number of vegetative primordia and subsequent FLN is determined from the HS timing of this event:
(5)
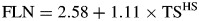



Once terminal spikelet occurs the crop will begin stem extension and grow out its remaining leaf organs to reach flag leaf and then anthesis.

### Relating anthesis genotype to phenotype

The model presented provides an explicit link between the time of anthesis and the Haun stage at which TS occurs. The occurrence of TS is the end result of a progression of gene expression (Fig. 8) and algorithms are presented to calculate the effects of environment on the expression of these genes. This suggests that a quantitative link between developmental genes and anthesis time can be created by determining relationships between the coefficients of this model and the alleles or copy numbers of genes that control the expression of protein signals.

There is considerable variation in vernalization responses of wheat and we propose these can be quantified by linking variation in *Vrn1* alleles and copy number to the BaseΔVrn1 coefficient (Eq. 2). This is consistent with observations of Diaz *et al.* (2012) that genotypes that saturate vernalization in a shorter time will have a faster ΔVrn1. A winter genotype would have an allele of Vrn1 that binds with repressors at high temperatures so BaseΔVrn1 would be small (0·08/HS for winter ‘Batten’) and [Vrn1] (Fig. 8) would accumulate slowly. Alternatively, spring wheat has an allele of Vrn1 that is less effective at binding with repressors so BaseΔVrn1 would be higher (0·19 for spring ‘Batten’) and [Vrn1] increases quickly at high temperatures. Thus, winter wheat would reach VS slowly and spring wheat would reach VS quickly at high temperatures. Under constant Pp the time from VS to TS will be constant with FLN closely related to TS^HS^ (Fig. 8). Thus, by holding different genotypes of wheat at 23 °C at 8-h Pp and counting their FLN one could determine their relative vernalization phenotypes and develop a method for relating this to BaseΔVrn1.

There is also variation in Pp sensitivity and we propose this may be quantified by linking alleles of *Ppd* and/or *Vrn3* to the difference between BaseΔ[Vrn3] and MUΔ[Vrn3] coefficients (Eq. 4). A genotype with little Pp sensitivity is ineffective at reducing Δ[Vrn3] in response to low Pp so would have a similar Δ[Vrn3] regardless of Pp. Low Pp would increase [Vrn3] (Fig. 8) and it would reach TS quickly. Alternatively, a genotype with a strong Pp response would have greater reduction of Δ[Vrn3] at lower Pp so would upregulate [Vrn3] slowly, take longer to reach TS and have a higher FLN. When a genotype of wheat is fully vernalized before emergence (by holding it at 1 °C for 100 d following imbibition), its final leaf number will be dependent on Δ[Vrn3]. So by fully vernalizing different genotypes and then exposing them to 8-h Pp conditions, the sensitive genotypes will have a higher FLN and the insensitive genotypes will have a low FLN, and a relationship could be constructed between this and MUΔ[Vrn3] − BaseΔ[Vrn3].

There is genetic variation in the extent of the vernalization response lag (Wang *et al.*, 1995*a*, *b*). We propose that this is due to variation in the *Vrn4* gene, such that genotypes that are missing the *Vrn4* gene will not express Vrn4 so are unable to repress the temperature response of *Vrn1*. They will not exhibit a vernalization lag as a result. Winter ‘Batten’ showed a long vernalization lag (Fig. 7A) so presumably has an active allele of *Vrn4* and started with a high initial [Vrn4] that had to be downregulated (Fig. 8). This meant it took longer for vernalization to begin, which then delayed the HS timing of all subsequent events and so made anthesis later. We propose the extent of this vernalization lag could be explained by linking each genotype's *Vrn4* alleles to the initial value of [Vrn4] and the MDΔ[Vrn4] coefficient (Eq. 1).

The importance of short-day vernalization is not well understood with regard to variation in anthesis time. However, this model provides a mechanism for linking short-day vernalization phenotype to variation in *Vrn2* alleles or copy number. For ‘Batten’ wheat, BaseΔ[Vrn2] and MUΔ[Vrn2] (Eq. 3) were zero and 0·028/HS, respectively. Genotypes with stronger short-day vernalization responses will have greater values of MUΔ[Vrn2] and so the [Vrn1] target (Fig. 8) will increase faster than for genotypes with weaker responses that will have smaller values of MUΔVrn2. Genotypes missing the *Vrn2* gene will have a MUΔVrn2 value of 0.

## CONCLUSIONS AND RECOMMENDATIONS

This paper presents a model that provides a framework for directly linking the genotype of a wheat plant to its time of anthesis in any environment, and novel methods of quantifying genetic and environment responses. The model is distinct from other models because it explicitly integrates notions of gene expression into the established physiological methods of quantifying anthesis time. This apporach removes the necessity for convenient mathematical fictions (such as a ‘potential’ leaf number that reduces with accumulating vernalization). Instead, all responses are to the direct environment and the current state of the organism.

The link between molecular biology and physiology is achieved through assuming that terminal spikelet (a key developmental event) occurs when the required gene expression levels have occurred. A second link is achieved by determining rates of gene expression relative to change in HS, which is predicted in response to Tt accumulation. Thus, gene-modulated development will increase with temperature per se.

It is clear from the analysis that vernalization involves responses to both temperature and Pp. These vernalization responses occur first and are completed before FI. Vernalization saturation is the expression of sufficient Vrn1 and this will eventually occur in all facultative wheat plants due to low base expression. When calculated as a function of HS, Vrn1 expression is increased most at 1 °C and decreases to the base rate at 23 °C. The winter isoline of ‘Batten’ showed a greater reduction in apparent Vrn1 expression at high temperature than the spring isoline. This provides a method for predicting genotype differences in low-temperature vernalization responses. The Vrn1 target for VS is dependent on levels of Vrn2 expression, with short days giving less Vrn2 expression, a lower Vrn1 target and faster vernalization. This mechanism provides a method for predicting short-day vernalization. The model assumes that Vrn4 must be downregulated to allow Vrn1 expression to increase above base rates and so causes a lag period where no vernalization response will occur. This provides a method for predicting vernalization response lags.

Following VS, Vrn3 is expressed and, when levels are sufficient, the crop will exhibit TS. The rate of Vrn3 expression increases with Pp. Thus, the pre- and post-VS responses to Pp are qualitatively different because long Pp allows faster Vrn3 expression after VS but long Pp also increases Vrn2 expression, which increases the vernalization target before VS.

The model provides a clear role for each of the known flowering time genes with *Vrn1* controlling low-temperature vernalization responses, *Vrn2* controlling short-day vernalization response, *Vrn3* and *PpD* controlling Pp responses, and *Vrn4* controlling vernalization lag responses. Further work is required to test the performance of this model by empirically deriving coefficients for the temperature and Pp response mechanisms from physiological observations and comparing anthesis date predictions with field observations. Should this be successful, we propose these coefficients can be estimated from the combination of *Vrn1*, *Vrn2*, *Vrn3*, *PpD*, *Vrn4* genotype. This will provide a method for quantifying the effect of genotype on the anthesis date of a specific genotype in a specific location. There is still a need for more detailed time course expression data to fully verify the gene expression components of this model, particularly in regard to Vrn4. There is also a need for further investigation into the genes that are controlling Pp responses in the ear development phase.
